# Metabolic orchestration driven by GGCT: diverting glutamine to glutathione biosynthesis while enhancing glucose anaplerosis for tumor proliferation

**DOI:** 10.1038/s41419-026-08619-y

**Published:** 2026-03-24

**Authors:** Lijun Yang, Handi Sun, Ruonan Wang, Depeng Yang, Qi Gu, Guiping Zhao, Liping Sun, Xinghe Chen, Jianxin Lv, Xiaoyu Lin, Jiahui Cheng, Muhammad Luqman Akhtar, Mengmeng Zhang, Jingyu Zang, Xinyue Shi, Zihao Zhang, Lijun Deng, Lixing Xiao, Lei Yue, Wei Dong, Qinghua Jiang, Fang Han, Yu Li, Huan Nie

**Affiliations:** 1 School of Life Science and Technology, Harbin Institute of Technology, Harbin, China; 2 National Key Laboratory of Immunity and Inflammation, Suzhou Institute of Systems Medicine, Chinese Academy of Medical Sciences & Peking Union Medical College, Suzhou, China; 3 Department of Hepatobiliary and Pancreatic Surgery, Harbin Medical University Cancer Hospital, Harbin, China

**Keywords:** Cancer metabolism, Cell growth

## Abstract

Glutamine (Gln) metabolism serves dual metabolic roles: it fuels the tricarboxylic acid (TCA) cycle, while concurrently sustaining redox balance through glutathione (GSH) synthesis. γ-Glutamylcyclotransferase (GGCT), a key metabolic enzyme frequently overexpressed in various cancers, has an undefined role in directing glutamine metabolic flux during tumorigenesis. This study demonstrated that glutamine promotes cancer cell growth by regulating GSH and reactive oxygen species (ROS) levels, with this process being closely associated with GGCT expression. Knockdown of GGCT significantly inhibited tumor growth, depleted GSH, and elevated ROS levels, whereas overexpression of GGCT exerted the opposite effects. Furthermore, we refined and established the Gln/c-Myc/miR-29b-3p/GGCT regulatory axis. Notably, GGCT knockdown markedly altered mitochondrial morphology and impaired oxidative phosphorylation and glycolysis capacity. Targeted metabolomics analysis revealed that GGCT knockdown significantly reduced the abundance of TCA cycle intermediates, while GGCT overexpression substantially increased their levels. [U-^13^C]glutamine isotope tracing experiments showed that GGCT overexpression reduced Gln contribution to the TCA cycle and diverted it preferentially to the GSH synthesis pathway for ROS regulation. In contrast, [U-^13^C]glucose isotope tracing results demonstrated a significant increase in TCA cycle intermediates derived from glucose when GGCT was overexpressed. Additional, supplementation of sodium pyruvate and JX06 in GGCT-knockdown cells confirmed that this regulatory effect of GGCT-mediated changes in ROS was independent of energy metabolism pathways. Collectively, this study identifies GGCT as a metabolic switch that diverts Gln flux toward GSH synthesis to maintain redox homeostasis, while enhancing glucose-fueled anaplerosis into the TCA cycle to sustain cell proliferation. These findings highlight GGCT as a potential therapeutic target for disrupting cancer redox adaptation and metabolic plasticity.

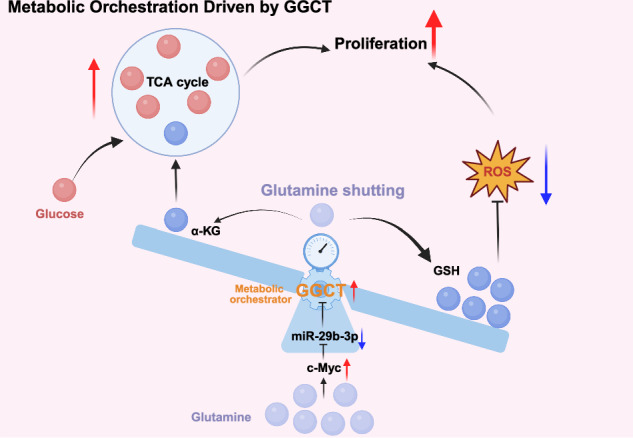

## Introduction

Central to metabolic reprogramming is the dysregulation of Glutamine (Gln) metabolism, which has emerged as a critical aspect of cancer pathophysiology. As a key energy substrate for tumor cells, Gln can be catabolized by glutaminase to generate α-ketoglutarate (α-KG), which directly enters the tricarboxylic acid (TCA) cycle [1]. Moreover, Gln and glucose metabolism form a tightly interconnected regulatory network that synergistically meets the energy and material demands of rapidly proliferating tumor cells, yet the underlying regulatory mechanisms remain largely elusive.

Gln also serves as a precursor for glutathione (GSH) synthesis to scavenge reactive oxygen species (ROS), thereby maintaining cellular redox homeostasis [2]. γ-Glutamylcyclotransferase (GGCT) is a key enzyme catalyzing 5-oxoproline formation in the γ - glutamyl cycle. As an important regulator in cancer metabolism, dysregulated GGCT expression has been confirmed to be closely associated with the progression of multiple malignancies [3–10]. Accumulating evidence has demonstrated that γ-glutamyl cyclotransferase (GGCT) exerts potent oncogenic effects across multiple cancer types via the modulation of diverse key signaling pathways. Specifically, GGCT has been confirmed to activate the PI3K/AKT/mTOR signaling, thereby driving the malignant progression of ovarian cancer, hepatocellular carcinoma, and breast cancer [11–13]. Beyond this classic signaling pathway that is widely implicated in tumorigenesis, GGCT could also regulate the Notch-Akt axis to facilitate the uncontrolled proliferation and aggressive growth of glioma cells [14]. Furthermore, GGCT formed a tightly regulated positive feedback loop together with REST and miR-34a-5p; this regulatory module could further amplify the pro-tumorigenic functions of GGCT and orchestrate the multi-step initiation and progression of glioma [10]. Although GGCT functions as a core metabolic enzyme, its regulatory role in directing Gln metabolic flux remains to be elucidated.

Here, our study elucidated the pivotal role of GGCT in driving hepatocellular cancer (HCC) and prostate cancer (PCa) progression by orchestrating Gln partitioning between redox homeostasis and the TCA cycle. We demonstrated that Gln promotes proliferation by maintaining GSH-ROS balance, and refined the Gln/c-Myc/miR-29b-3p/GGCT regulatory axis, which links Gln availability to GGCT expression. Further, targeted metabolomics and isotope tracing experiments uncovered GGCT as a metabolic orchestrator to prioritize Gln to GSH flux for antioxidant defense, while activating glucose (Glc)-fueled anaplerosis to replenish TCA intermediates to fuel proliferation. Thus, the discovery positions GGCT as a promising therapeutic target for disrupting metabolic plasticity through its dual roles in Gln shunting and Glc compensation.

## Materials and methods

### Clinical samples of hepatocellular carcinoma patients

Human hepatocellular carcinoma (HCC) cancer tumor tissues and matched adjacent tissues (*n* = 20) were collected from Cancer Hospital Affiliated to Harbin Medical University for qRT- PCR, western blot and Eliza assays. Both HCC and adjacent tissues have been histologically confirmed. Informed consent was obtained from all enrolled participants in the study. The research protocol was performed with the approval of the Ethics Committee of Harbin Institute of Technology (Approval No: HIT-2022008).

### Cell culture

Human HCC cell lines MHCC97H, HepG2 and PCa cell line DU145 were cultured in high-glucose DMEM complete medium with 10% fetal bovine serum (FBS; Gibco) at 37 °C and 5% carbon dioxide. Human prostate cancer (PCa) cell lines LNCaP and C4-2 were maintained in RPMI 1640 medium supplemented with 10% FBS. PCa cell lines PC3 were cultured in Ham’s F-12 K complete medium. All cell lines were obtained from the American Type Culture Collection (ATCC).

### Plasmid construction and siRNA

Plvsin-GGCT-eGFP expression plasmid was constructed by inserting the full-length GGCT sequence amplified from human cDNA and ligated into the Plvsin-eGFP vectors. Plvsin-GGCT E98A-eGFP plasmid was modified by mutating the glutamate (E) at position 98 into alanine (A) using Plvsin-GGCT-eGFP plasmid. All plasmids were verified by DNA sequencing (COME company, Jilin, China).

The siRNAs (siNC, siGGCT162, siGGCT299) were synthesized in GenePharma Company (Shanghai, China). The PCR amplification primers and siRNA sequences were provided in Supplementary Table S1.

### Cell viability assay

For cell viability assay, cells were planted in 96-well plates (3–4 × 10^3^ per well) after indicated treatment, then incubated with 10% CCK-8 reagent at 37 °C for 2 h. Absorbance was measured at 450 nm. For cell colony formation assay, cells were seeded in 6-well plated at a density of 1–2 × 10^3^ cells per well. After 15 days of culture under different treatment, the visible colonies were formed. The plates were washed with PBS three times, and stained with 0.1% crystal violet. Then, the colonies were counted by using Image J and each experiment was repeated three times.

### Quantitative real-time PCR (qRT-PCR) and Western blot assay

Total RNA was extracted using Trizol reagent (Invitrogen) according to the manufacturer’s instructions protocol. CDNA was conducted by using Transcriptor Reverse Transcriptase (Takara, RR036A). The real-time PCR reaction was carried out using a SYBR Green PCR kit (Takara, RR820). The relative levels of targeted gene expression were measured by the 2^−ΔΔ CT^ method. The primer sequences were provided in Supplementary Table S2.

Total proteins from lysed cells or patient tissue were extracted using RIPA lysis buffer and electrophoresed by SDS-PAGE and transferred onto PVDF membranes subsequently. The membranes were incubated with 5% BSA at room temperature for 1 h, then with primary antibodies at 4 °C overnight and second antibody successively for 1 h at room temperature, and consequently developed on the imaging system after adding ECL reagent. The antibody information was listed in Supplementary Table S3.

### Immunohistochemical staining (IHC)

A HCC tissue microarray containing 10 pairs of tumor tissues and matched adjacent tissues (HLivH020PG01) and a PCa tissue microarray containing 3 normal tissues, 8 adjacent tissues and 49 tumor tissues (HProA060PG01) were purchased from Outdo Biotech (Shanghai, China). After deparaffinization and hydration, the sections were treated with heated citrate buffer (pH 6.0), 0.3% H_2_O_2,_ and blocked with 3% BCA successively, then incubated with primary antibody at 4 °C overnight and biotinylated secondary antibody. At last, the sections were colored with 3, 5-diaminobenzidine substrate, and then counterstained with hematoxylin.

The scoring standard for IHC staining adopted the immunoreactive score (IRS) system [15, 16], with the specific rules as follows: the staining intensity of GGCT protein was classified into four grades, where a score of 0 indicated no staining signal, 1 indicated weak positivity, 2 indicated moderate positivity, and 3 indicated strong positivity; the proportion of positively stained cells was divided into four grades, namely (1) 0–25%, (2) 26–50%, (3) 51–75%, and (4) 76–100%; the final immunoreactive score for each tumor specimen was calculated by multiplying the staining intensity score by the positive cell percentage score; the staining intensity grading of all cases was independently and blindly repeated three times by two investigators to ensure the objectivity and reliability of the scoring results.

### ROS detection

Intracellular production of ROS was measured using DCFH-DA prob. To determine ROS production, cells were incubated with DCFH-DA (10 μM) for 30 min, washed twice with cold PBS. ROS production was analyzed by a flow cytometry.

### Chromatin immunoprecipitation (CHIP)

CHIP assays were carried out strictly in accordance with the manufacturer’s operational guidelines (Qiagen, Dusseldorf, Germany). Briefly, cells pretreated with 4 mM glutamine (Gln) and 0 mM Gln, respectively, were cross-linked with formaldehyde at 37 °C for 10 min, followed by the addition of glycine solution to quench the cross-linking reaction. Next, the lysis buffer was added to disrupt cell membranes and release intracellular constituents. Chromatin DNA was subjected to mechanical fragmentation via sonication, and the resulting protein-DNA complexes were immunoprecipitated using an anti-MYC antibody. After isolation of the target DNA–protein complexes, Proteinase K was added to degrade the protein components within the complexes, and the purified DNA products were recovered. Finally, the purified DNA samples were subjected to reverse transcription-quantitative polymerase chain reaction analysis and high-throughput sequencing.

### Mitochondrial morphology analysis

The mitochondrial morphology of HCC and PCa cells was evaluated by transmission electron microscopy (FEI, Hillsboro, Oregon, USA) as described previously. Image J software (NIH, Bethesda, MD, USA) was used for analysis of mitochondria length.

### Metabolic analysis

Oxygen consumption rate (OCR) and extracellular acidification rate (ECAR) were measured with a 24-well XF Extracellular Flux Analyzer (Agilent Technologies) and Seahorse Wave Controller Software (Agilent Technologies), according to the manufacturer’s instructions. OCR analysis was performed using the Cell Mito Stress Test Kit, while ECAR analysis relied on the Glycolysis Stress Test Kit (Agilent Technologies). Briefly, the treated cells were seeded at a precise density for overnight culture, washed with basic medium, and equilibrated at 37 °C for 1 h. Subsequently, the respective reagents from the OCR and ECAR assay kits were added to the cells. After a 20 min incubation, measurements were performed using a Seahorse XF24 Extracellular Flux Analyzer.

### Luciferase reporter assay

A luciferase reporter assay was assessed as previously described with modifications [17]. Briefly, to verify the targeted binding between miR-29b-3p and the GGCT gene, a 2000-bp DNA fragment (derived from the transcription start site of the GGCT gene) was inserted into the pmir-GLO-Basic vector to construct the wild-type pmir-GLO-GGCT-3′ UTR reporter plasmid (wt) and the mutant pmir-GLO-GGCT-3′ UTR reporter plasmid (mut). The miR-29b-3p mimics and inhibitor were synthesized by GenePharma (China), and the relevant sequence information is provided in Supplementary Table S4. To investigate the transcriptional regulatory effect of Gln on the GGCT gene, a 2000-bp sequence of the GGCT gene promoter region was inserted into the pGL4-Basic vector. The recombinant vectors were then transfected into cells, which were cultured in medium containing 4 mM or 0 mM Gln, respectively, followed by detection of luciferase activity. For all the aforementioned experiments, the Dual-Luciferase Reporter Assay System (Promega Corporation) was used to measure luciferase activity strictly according to the manufacturer’s instructions.

### Animal experiments

Four-week-old male BALB/C nude mice were purchased from the animal center of Changsheng Biotechnology (Liaoning, China), and kept in a specific pathogen-free condition and randomly divided into groups. For evaluation of in vivo tumor growth, a total of 6 × 10^5^ cells (resuspended in 50 μL DMEM medium and 50 μL Matrigel) were subcutaneously injected into the flank of mice. Tumor volumes (0.52 × length × width^2^) [18] and mice weight were measured every 5 days for 20 days. Mice were euthanized by cervical dislocation, and the tumor were removed for weighing and volume measurement at endpoint. Blinding was not performed during animal experiments and data collection, but outcome assessments (e.g., IHC scoring) were conducted by blinded investigators. All mice were approved by the Laboratory Animal Welfare Ethics committee at Harbin Institute of Technology (Approval No: IACUC- 2024138) and conducted in accordance with the Guide for Care and Use of Laboratory Animals and the institutional ethical guidelines for animal experiments.

### Bioinformatics analysis

PCa gene expression datasets (GSE148016 and GSE154584) were obtained from the GEO database (http:// www. ncbi. nlm. nih.gov/ geo/). The RNA-seq data on knockdown of GGCT in the MHCC97H cell have been submitted to the National Center for Biotechnology Information (http:// www. ncbi. nlm. nih.gov/ geo/), with the ID number GSE282603. The “limma” R software package (adjusted *p* < 0.05 and |log_2_ fold change (FC)| > 1) was used to screen differentially expressed genes (DEGs). The “ggplot2” and “pheatmap” R software packages were conducted to visualize the DEGs by drawing volcano plots and heatmaps, respectively. GSEA enrichment analysis was performed using the “fgsea” software package based on the FC values of sorted genes. The expression correlation between genes was calculated by Pearson’s correlation coefficient, and a network diagram was constructed using “cytoscape”. Furthermore, the Gene Ontology (GO) and KEGG pathway enrichment analysis were processed through the Database for Annotation, Visualization and Integrated Discovery (DAVID; http://david.ncifcrf.gov).

### LC-MS-based metabolomics and metabolic flux analysis

Sample preparation was as follows. Cells were extracted with 200 μL of ice-cold 80% methanol, centrifuged at 15,000 *g* for 20 min at 4°C and lyophilized. Subsequently, added 60 μL of 50% acetonitrile, vortexed with shaking at 1700 rpm for 10 minutes at 8 °C, centrifuged at 15,000 *g* for 20 minutes at 4 °C, and aspirated the supernatant and stored at −80 °C until analysis. Sample analysis was performed on an Agilent 1290 UPLC system equipped with a Waters BEH amide (2.1 mm × 100 mm, 1.7 μm) column using a gradient of 15 mM NH₄Ac/0.3% NH₄OH in H₂O (A) and 90% ACN (B) at 0.3 mL/min. The gradient elution program was as follows: from 0 to 7 minutes, the 90% B was decreased to 65%; from 7 to 7.5 min, Phase B was further decreased to 50%; then, 50% Phase B was maintained for 2.5 minutes, and the post-run time was 7 min. The injection volume was 2 μL. Data acquisition was using an Agilent 6546 Q-TOF instrument (ESI±) with optimized parameters: gas temp 300 °C, drying/sheath gas 8/11 L/min, capillary voltage 3500 V, and spray voltages ±500/1000 V. Data were processed using Profinder 10.0 software.

### Statistical analysis

All the statistical analyses were performed with GraphPad Prism 10.0 software (GraphPad Software, San Diego, CA). The data are presented as mean ± SD from three independent experiments. Student’s *t* test and One-way ANOVA test were used for statistical analysis. Pearson and Spearman correlation analyses were used to estimate the correlation between measured variables. The probability of survival was estimated using the Kaplan-Meier method. Statistically significant differences are represented in figures as * for *p* < 0.05, ** for *p* < 0.01 and *** *p* < 0.001, respectively.

## Results

### Gln drives cellular proliferation by dynamically regulating the intracellular redox balance

**Fig. 1:**
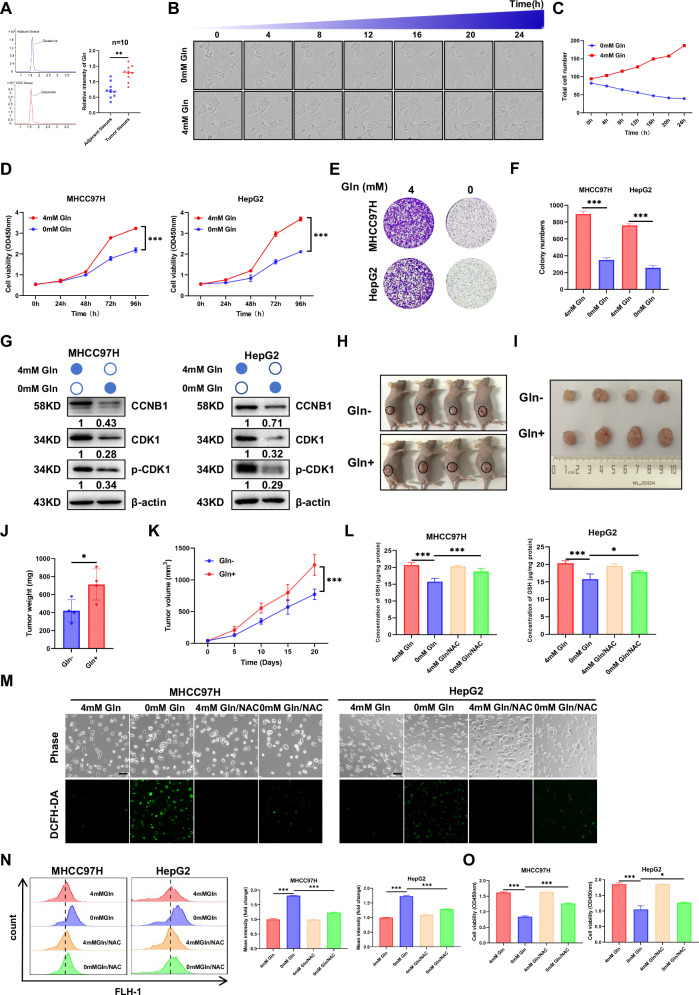
Gln promoted proliferation, affected ROS and GSH levels in HCC cells. **A** Representative extracted ion chromatograms (EICs) and comparative intensity bar plots of Gln in HCC and adjacent tissues (*n* = 10). The upper left panel shows a representative mass spectrum of Gln in adjacent normal tissues; the lower left panel shows a representative mass spectrum of Gln in HCC tissues; the right panel is a bar graph depicting the quantitative comparison of Gln levels between the 10 pairs of HCC tissues and adjacent normal tissues. **B**, **C** Representative phase-contrast images of MHCC97H cells treated with Gln- and Gln+ conditions for 24 h, with corresponding time-course quantification of total cell numbers. The upper row shows the dynamic growth profiles of cells monitored over time under Gln⁻ conditions; the lower row shows the dynamic growth profiles of cells monitored overtime under Gln⁺ conditions. All figures presented correspond to magnified partial regions of the original samples. **D** The CCK-8 assay was conducted in MHCC97H and HepG2 cells treated with 0 mM and 4 mM Gln. **E**, **F** The cell colony formation assay was performed in MHCC97H and HepG2 cells under 0 mM and 4 mM Gln culture conditions. **G** The western blot analysis was applied to detect the protein level of CCNB1, CDK1 and p-CDK1 in MHCC97H and HepG2 cells treated with 0 mM and 4 mM Gln. **H**–**I** Tumors from sacrificed mice developed from MHCC97H cells with Gln deprivation or sufficiency treatment were dissected 21 days after transplantation. **J** Tumor weights of subcutaneous xenograft tumor model developed from MHCC97H cells with Gln deprivation or sufficiency treatment were measured after removal. **K** Tumor growth curves developed from MHCC97H cells with Gln deprivation or sufficiency treatment (*n* = 4). Tumor size including tumor length (*L*) and width (*W*) was measured using vernier calipers every 5 days from second week after transplantation. **L** The GSH levels of MHCC97H and HepG2 cells treated with 0 mM and 4 mM Gln with or without N-acetylcysteine (NAC) as an antioxidant. **M** Representative images of ROS detection in MHCC97H and HepG2 cells under different Gln conditions, with or without NAC treatment. Scale bar:100 µm. **N** Relative fluorescence of DCFH-DA staining for ROS detection in MHCC97H and HepG2 cells under different Gln conditions, with or without NAC treatment in flow cytometer. **O** The cell viability of MHCC97H and HepG2 cells treated with 0 mM and 4 mM Gln, with and without NAC. (Data represent means ± SD. ns: not significant, *p < 0.05, **p < 0.01, and ***p < 0.001).

Preliminary metabolomic results revealed that Gln metabolism was significantly enriched in HCC [19]. In this regard, the level of Gln was analyzed using mass spectrometry and was found to be higher in HCC tissues than in adjacent tissues (Fig. 1A). Live cell imaging results showed that some cells exhibited a state of non-division or nonextension under Gln deficiency (Gln−) conditions, whereas cells grew plum and well under Gln sufficiency (Gln+) conditions (Fig. 1B, C). Furthermore, we analyzed the differences in the PCa cell under Gln deprivation (0 mM Gln) and repletion (4 mM Gln) using the published dataset GSE148016. Functional enrichment showed that KEGG and GO (BP) were both focused on the cell cycle (Fig. S1A, B). Moreover, the cell proliferation ability of HCC cell lines (MHCC97H and HepG2) and PCa cell lines (LNCaP and C4-2) increased with an increase in Gln concentration, as determined by cell CCK- 8 and colony formation assays (Fig. 1D–F; Fig. S1C–E). Notably, Gln deprivation significantly reduced the expression of CCNB1, CDK1 and p-CDK1 in HCC and PCa cells (Fig. 1G; Fig. S1F). Additionally, xenograft tumor data showed that Gln significantly promoted tumor growth in vivo (Fig. 1H–K; Fig. S1G, H). Owing to the involvement of Gln in GSH synthesis, we detected changes in intracellular GSH and reactive oxygen species (ROS). The results illustrated that Gln restriction led to a pronounced decline in intracellular GSH levels (Fig. 1L; Fig. S1I), accompanied by elevated ROS (Fig. 1M, N; Fig. S1J, K). Administration ofthe ROS inhibitor N- acetylcysteine (NAC) partially restored GSH, attenuated ROS accumulation, and modestly alleviated Gln-deprivation-induced growth inhibition (Fig. 1O; Fig. S1L). These results suggest that Gln can regulate cell growth, which partially originates from the maintenance of the redox balance in tumor cells.

### GGCT expresses higher and correlates positively with Gln in a dose-dependent manner in tumor cells

**Fig. 2:**
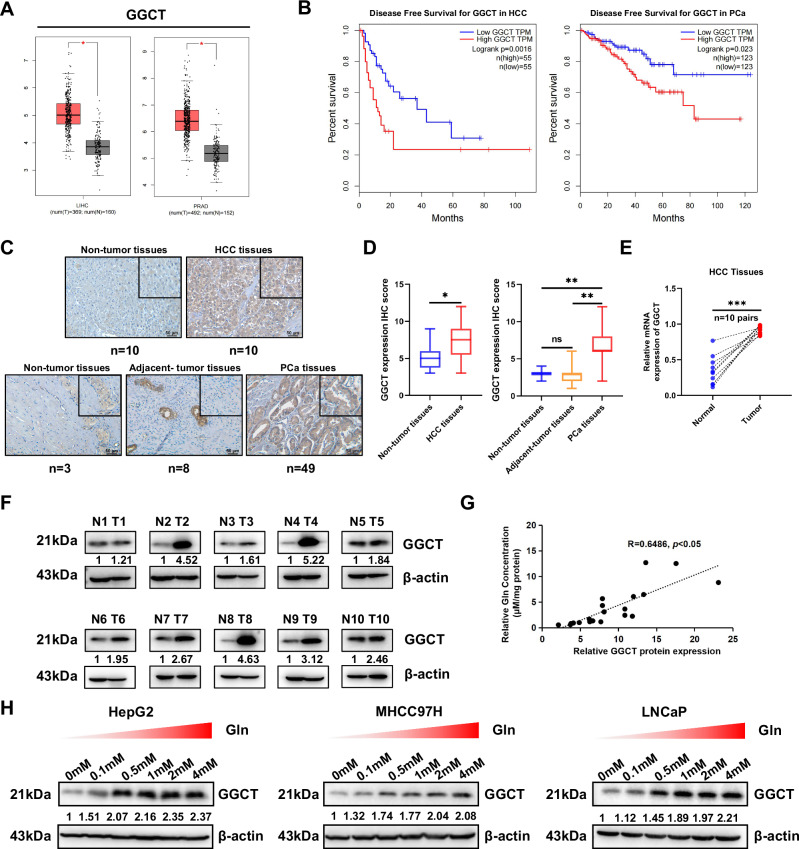
GGCT is upregulated in PCa and HCC and correlates with Gln in a dose-dependent manner. **A** Box plots showing GGCT expression levels in HCC and PCa tissues versus non-tumor tissues from the TCGA dataset. **B** Kaplan-Meier survival curves for disease-free survival (DFS) stratified by GGCT expression in PCa and HCC patients grouped by the expression of GGCT in tumor tissues. **C** Immunohistochemistry (IHC) images showing GGCT protein expression in normal tissues and HCC tissues (top row) as well as in normal tissues, adjacent tissues, and PCa tissues (bottom row). Scale bar: 50 µm. **D** IHC score of GGCT in HCC and PCa tissues compared to nontumor tissues and adjacent tissues. Data are presented as mean ± SD, with significant differences indicated. **E** Relative mRNA expression levels of GGCT in paired normal and tumor tissues from HCC patients. **F** Western blot analysis of GGCT protein levels in paired nontumor (N) and tumor (T) tissues from HCC patients. **G** Correlation analysis between GGCT protein expression and Gln concentration in HCC tissues. **H** Western blot analysis of GGCT protein expression in HepG2, MHCC97H, and LNCaP cells treated with varying concentrations of Gln (0, 0.1, 0.5, 1, 2, and 4 mM) (Data represent means ± SD. ns: not significant, *p < 0.05, **p < 0.01, and ***p < 0.001).

Given Gln’s role in GSH synthesis, we analyzed GSH metabolism gene expression in HCC and PCa using the GEPIA database. Among these genes, only GGCT expression was significantly elevated in tumor tissues compared to normal tissues both in HCC and PCa, whereas other genes (GCLC, GCLM, GGT1, and others) showed no significant increase (Fig. 2A; Fig. S2A). Analysis of TCGA database revealed that GGCT is overexpressed in most tumors (Fig. S2B). Clinically, disease-free survival results confirmed that poor outcomes were associated with upregulation of GGCT expression in HCC and PCa (Fig. 2B). Immunohistochemistry (IHC) in HCC and PCa, as well as mRNA and protein detection in clinical HCC samples, confirmed significantly higher GGCT protein levels in tumor tissues versus adjacent non-tumorous or normal tissues (Fig. 2C–F). Strikingly, the concentration of Gln was positively correlated with GGCT protein expression in HCC tissues (Fig. 2G). Furthermore, we observed that GGCT was highly expressed in a dose-dependent manner in HCC and PCa cell lines both treated for 24 h (Fig. 2H). These results suggest that GGCT is highly expressed in tumors and that its expression is positively regulated by Gln in a dose-dependent manner.

### Gln inhibits miR-29b-3p transcription via c-Myc regulation to promote GGCT protein expression

**Fig. 3:**
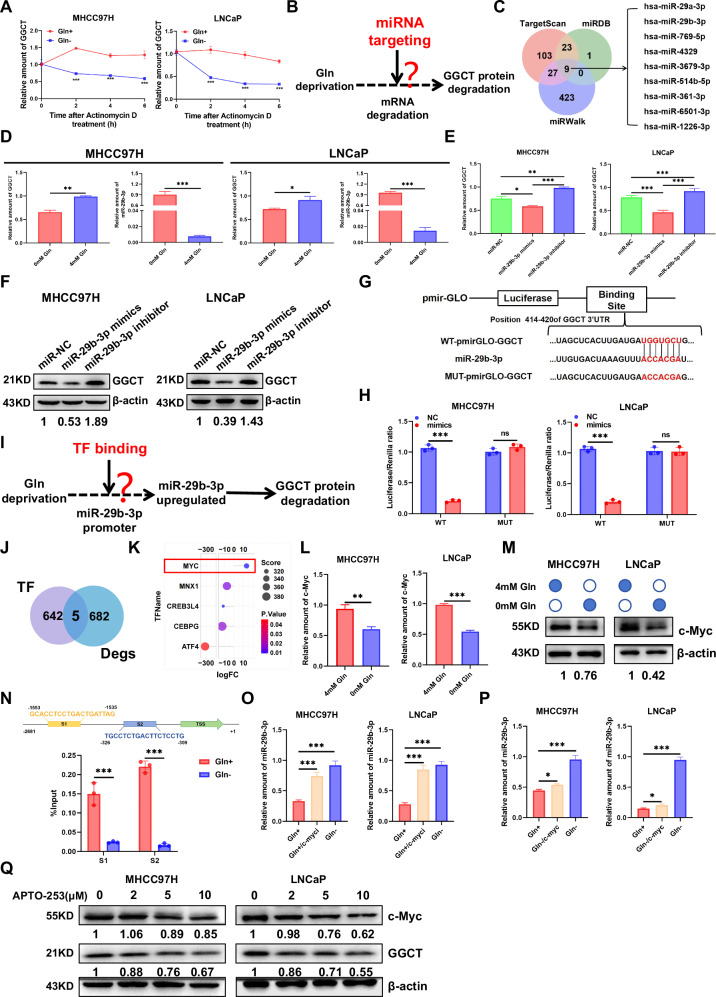
Gln inhibits miR-29b-3p transcription via c-Myc regulation to promote GGCT protein expression. **A** Stability of GGCT mRNA treated with Gln+ and Gln- conditions in MHCC97H and LNCaP cells. **B** Proposed model depicting the molecular mechanism of reduced expression of GGCT protein under Gln deficiency conditions. **C** Venn diagram displaying the overlap of miRNAs predicted to target GGCT mRNA according to TargetScan, miDB, and miRWalk databases. **D** qRT-PCR detected the expression of GGCT mRNA and miR-29b-3p cultured with Gln- and Gln+ both in MHCC97H and LNCaP cells. **E** Relative expression levels of GGCT mRNA in MHCC97H and LNCaP cells transfected with miR-29a-3p mimics and inhibitor or negative control (NC). **F** Western blot analysis of the effect of miR-29b-3p mimics or inhibitors on GGCT protein levels in MHCC97H and LNCaP cells. **G** Schematic representation of the binding sites of miR-29b-3p and the expression vectors construction of the wild type and mutant GGCT- 3’-UTR pmir-GLO plasmids used in luciferase reporter assays. **H** The relative luciferase activity was measured and the values were normalized to Renilla luciferase activity both in MHCC97H and LNCaP cells, cotransfected with WT-pmirGLO-GGCT (wt) or MUT-pmirGLO-GGCT (mutant) with NC or miR-29b-3p mimics. **I** Proposed model depicting the molecular mechanism by which Gln activates miR-29b-3p. **J** Venn diagram of transcriptomic data from Gln⁺ and Gln^−^ groups and predicted miR-29b-3p promoter. **K** Bubble chart of differential expression of five candidate transcription factors. **L**, **M** c-Myc mRNA and protein expression in MHCC97H and LNCaP cells under Gln⁺ and Gln⁻ conditions. **N** CHIP-qPCR detection of the 2 binding sites (S1 and S2) of c-myc to the miR-29b-3p promoter region between Gln+ and Gln- conditions in MHC97H and LNCaP cells. **O** Quantitative analysis of miR-29b-3p in Gln⁺, Gln⁻ and c-Myc-suppressed Gln⁺ groups by qPCR. **P** qRT-PCR analysis of miR-29b-3p expression Quantitative analysis of miR-29b-3p in Gln⁺, Gln⁻ and c-Myc-overexpressed Gln⁻ groups in MHC97H and LNCaP cells. **Q** Detection of GGCT protein expression following treatment with the c-Myc inhibitor APTO-253. (Data represent means ± SD. ns: not significant, *p < 0.05, **p < 0.01, and ***p < 0.001).

To determine GGCT downregulation during Gln deprivation, we first excluded protein degradation pathways as proteasomes (MG132) or lysosomes (CQ) inhibition failed to restore GGCT levels (Fig. S3A). Meanwhile, Luciferase activity assays revealed no significant difference in GGCT promoter activity between Gln+ and Gln- groups, indicating that Gln does not directly regulate GGCT transcription (Fig. S3B). Subsequently, we performed mRNA stability assays by treating cells with actinomycin D and found that GGCT mRNA degradation rate was significantly accelerated under 0 mM Gln compared with 4 mM Gln, indicating that Gln regulates GGCT in a post-transcriptional manner (Fig. 3A). Therefore, we hypothesized whether GGCT expression is mediated by miRNAs (Fig. 3B). Integration of target predictions from TargetScan, miRWalk, and miRDB identified nine potential miRNAs that target GGCT (Fig. 3C). Among these candidates, miR-29b-3p and miR-769-5p showed significantly altered expression in HCC and PCa tissues compared with adjacent non-tumor tissues (Fig. S3C). However, no significant correlation was detected between miR-769-5p and GGCT, leading to its exclusion from subsequent analyses (Fig. S3D). Moreover, evidence has shown that miR-29b-3p is a tumor suppressor factor in various cancers; therefore, subsequent research should mainly focus on the expression of miR-29b-3p. qPCR confirmed miR-29b-3p was downregulated in HCC tissues, inversely correlating with elevated GGCT (Fig. S3E). Gln deprivation significantly increased miR-29b-3p levels in MHCC97H and LNCaP cells, negatively correlating with GGCT expression (Fig. 3D). Next, to investigate whether miR-29b-3p regulates GGCT expression, a transfection with miR-29b-3p mimics and inhibitor was performed, and the transfection efficiency was verified (Fig. S3F). Transfecting cells with a miR-29b-3p mimics decreased GGCT mRNA and protein, while a miR-29b-3p inhibitor increased GGCT levels (Fig. 3E, F). We observed a significant decrease proliferation in miR-29b-3p mimics group, while increase in miR-29b-3p inhibitor group compared with the NC group (Fig. S3G). Furthermore, a dual luciferase reporter system assay demonstrated that the luciferase activity of GGCT-3′ untranslated region (UTR) (wild-type) decreased after transfection with miR-29b-3p mimics, while the luciferase activity of GGCT-3′ mUTR (mut) did not show significant changes in MHCC97H and LNCaP cells (Fig. 3G, H). In summary, these findings reveal a previously unrecognized miR-29b-3p-mediated regulatory circuit linking Gln availability to GGCT expression.

To dissect the upstream regulatory mechanism of miR-29b-3p under Gln deprivation conditions, we integrated transcriptome data (GSE148016) from both Gln+ and Gln- groups, and performed cross-analysis with the predicted upstream transcription factors of miR-29b-3p, ultimately identifying five candidate transcription factors. Among these factors, c-Myc exhibited the most significant differential expression, and subsequent experimental validation further confirmed that c-Myc was markedly downregulated under Gln deprivation (Fig. 3J–M). Based on potential binding regions predicted by JASPAR database, we designed specific primers targeting the binding site between c-Myc and the miR-29b-3p promoter (Table S2). Results from subsequent ChIP assays demonstrated that Gln deprivation significantly inhibited the binding of c-Myc to the miR-29b-3p promoter (Fig. 3N). qRT-PCR results revealed that the expression of miR-29b-3p was significantly downregulated under Gln+ culture conditions, whereas inhibition of c-Myc reversed this downregulation trend. In contrast, under Gln-deprived conditions, miR-29b-3p expression was markedly upregulated, while c-Myc overexpression completely abrogated this upregulation effect (Fig. 3O, P). Collectively, these results confirm that c-Myc is a key inhibitory mediator linking Gln deprivation to the regulation of miR-29b-3p expression. On this basis, we further investigated the impact of c-Myc suppression on GGCT expression, and the results showed a significant reduction in GGCT protein levels (Fig. 3Q). Taken together, these results revealed Gln regulates GGCT expression by modulating c-Myc-mediated transcriptional repression of miR-29b-3p.

### GGCT affects the proliferation by disrupting the redox balance in tumor cells

**Fig. 4:**
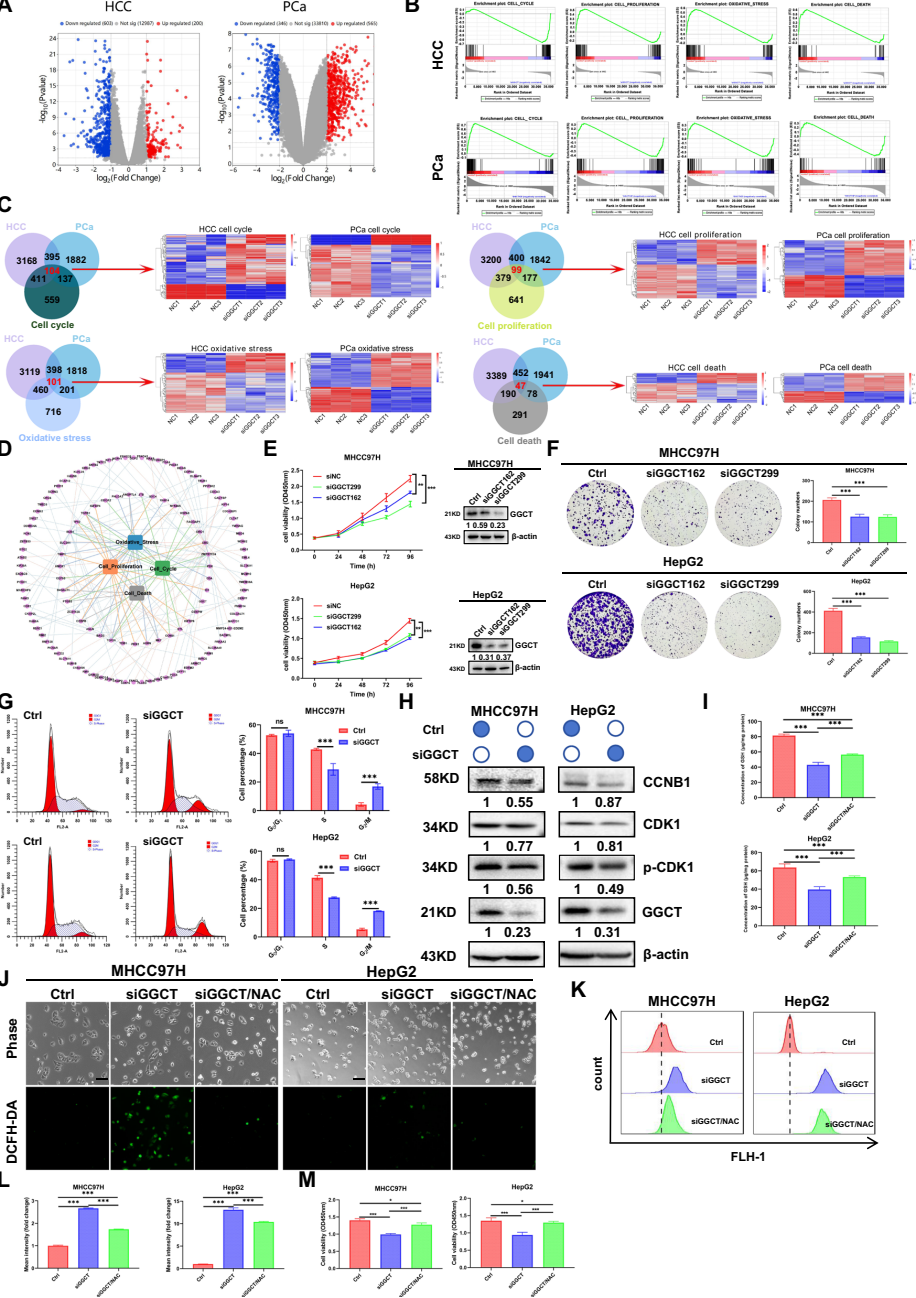
GGCT affects the proliferation by disrupting the redox balance in tumor cells. **A** Volcano plots showing differentially expressed genes in HCC and PCa datasets. Red and blue dots represent significantly upregulated and downregulated genes, respectively. **B** Gene Set Enrichment Analysis (GSEA) plots for HCC and PCa, highlighting pathways enriched in GGCT-high expression samples, including cell cycle progression, proliferation, and oxidative stress pathways. **C** Venn diagrams and heatmaps showing the overlap of differentially expressed genes related to cell cycle, proliferation, oxidative stress, and cell death in HCC and PCa. The heatmaps illustrated the expression patterns of these genes in relation to GGCT levels, with upregulated and downregulated genes indicated in red and blue, respectively. **D** Network diagram of GGCT and its interacting proteins, illustrating pathways associated with cell cycle, proliferation, oxidative stress and cell death. Nodes represent proteins, and edges represent interactions, with key pathways highlighted in colored clusters. **E** Cell proliferation assays in MHCC97H and HepG2 cells following GGCT knockdown. Western blot analysis confirms GGCT knockdown, with β-actin as a loading control. **F** Colony formation assays in MHCC97H and HepG2 cells with GGCT knockdown. Quantitative analysis on the right shows a significant reduction in colony number upon GGCT knockdown. **G** Cell cycle distribution detected by flow cytometry in GGCT-knockdown MHCC97H and HepG2 cells. **H** Western blot analysis of cell cycle-related proteins (CCNB1, CDK1 and p-CDK1) in MHCC97H and HepG2 cells after GGCT knockdown. **I** Quantification of GSH levels in MHCC97H and HepG2 cells following GGCT knockdown and pretreatment with NAC, respectively. **J** Representative images of DCFH-DA staining for ROS detection in MHCC97H and HepG2 cells after GGCT knockdown and pretreatment with NAC, respectively. Scale bar: 100 µm. **K**, **L** Quantification of DCFH-DA staining intensity, demonstrating increased ROS production following GGCT knockdown and pretreatment with NAC in both cell lines. **M** Cell viability of knocking down GGCT and pretreatment with NAC. (Data represent means ± SD. ns: not significant, *p < 0.05, **p < 0.01, and ***p < 0.001).

To explore the biological function of GGCT, we analyzed transcriptome data by knocking down GGCT in MHCC97H cells (GSE282603) and compared it with the published data from PC3 cells (GSE154584). The volcano plot displayed the up- and downregulated differential genes in each dataset (Fig. 4A). Functional enrichment (GO/KEGG) and Gene Set Enrichment Analysis (GSEA) revealed that GGCT knocking down significantly impacted pathways related to the cell cycle, oxidative stress, cell proliferation, and cell death in both cancers (Fig. 4B; Fig. S4A, B). Furthermore, Hierarchical clustering and network analysis confirmed these pathways were central to the differentially expressed genes (DEGs) (Fig. 4C, D). The results of proliferation assays indicated that HCC and PCa cells treated with siGGCT drastically inhibited cell proliferation compared with the control groups (Fig. 4E, F; Fig. S4C, D). Conversely, GGCT overexpression rescued proliferation under Gln deprivation in PC3 cells (Fig. S4C, D). As shown in Fig. 4G, H and Fig. S4E, F, depletion of GGCT in HCC and PCa cells led to G_2_/M phase arrest and decreased CCNB1 and CDK1 and p-CDK1 protein levels. We further found that knockdown of GGCT significantly reduced the GSH concentration in HCC and PCa cells and increased ROS production using DCFH-DA fluorescence detection (Fig. 4I–L; Fig. S4G–J). The addition of NAC increased the concentration of GSH and alleviated ROS production. Additionally, NAC pretreatment attenuated the cell viability induced by GGCT knockdown (Fig. 4M; Fig. S4K). Overexpression of GGCT in the presence of Gln significantly increased GSH and inhibited ROS production (Fig. S4L, M). Furthermore, GGCT overexpression reversed the inhibition of miR-29b-3p both in MHCC97H and LNCaP cells (Fig. S4N). These results suggest that GGCT promotes HCC and PCa cell proliferation partially by regulating intracellular redox balance (GSH/ROS).

### GGCT channels Gln flux for GSH synthesis and Glc anaplerosis into the TCA cycle

**Fig. 5:**
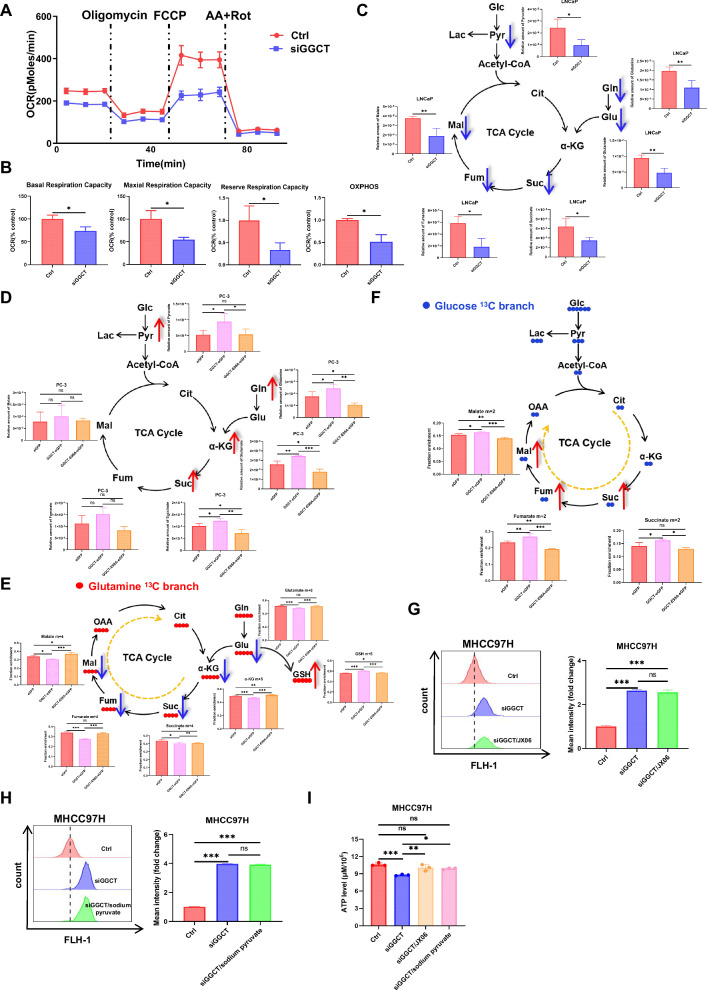
GGCT reprograms mitochondrial metabolism by redirecting glutamine/glucose flux into the TCA Cycle. **A** Detection of oxygen consumption rate (OCR) in GGCT-knockdown cells. Oligomycin, FCCP and Rot/AA were added sequentially as indicated by dashed lines. The OCR measurements were normalized to total cell numbers. **B** Basal mitochondria respiratory capacity, maximal mitochondria respiratory capacity, reserved mitochondria respiratory capacity and OxPhos capacity in siNC and siGGCT cells. **C** Schematic diagrams of the TCA cycle showing key metabolites affected by GGCT knockdown. Bar plots below each metabolite indicate relative levels in control and experimental groups. **D** Schematic diagrams of the TCA cycle showing key metabolites affected by GGCT overexpression. Bar plots below each metabolite indicate relative levels in control and experimental groups. **E** Schematic showing how [U-^13^C]glutamine carbons are metabolized through pathways of the TCA cycle and GSH in PC3 cells stable with GGCT and GGCT(E98A) mutant. **F** Schematic showing how [U-^13^C]glucose carbons are metabolized pathways of the TCA cycle and the fractional enrichment in PC3 cells stable with GGCT and GGCT(E98A) mutant. **G** Analysis of ROS levels via flow cytometry in control GGCT-knockdown, and GGCT-knockdown supplemented with sodium pyruvate. **H** Analysis of ROS levels via flow cytometry in control, GGCT-knockdown, and GGCT-knockdown tumor cells supplemented with JX06. **I** Measurement of intracellular ATP levels in control, GGCT-knockdown, GGCT-knockdown with sodium pyruvate supplementation, and GGCT-knockdown with JX06 treatment tumor cell (Data represent means ± SD. ns not significant, *p < 0.05, **p < 0.01, and ***p < 0.001).

ROS act as signaling molecules that regulate mitochondrial function, with mitochondrial morphology intrinsically linked to core processes, including the TCA cycle and oxidative phosphorylation. GGCT knockdown induced extensive mitochondrial elongation in C4-2 and HepG2 cells using transmission electron microscopy (TEM), suggesting the abnormal mitochondrial function (Fig. S5A, B). Consistently, GGCT knockdown reduced mitochondrial respiratory capacity (basal, maxillary and reserve respiration) in C4-2 cells compared with the control group (Fig. 5A, B). In contrast, GGCT knockdown led to a significant increase in the extracellular acidification rate (ECAR) (Fig. S5C, D). Further, targeted metabolomics results revealed that GGCT knockdown markedly attenuated TCA cycle flux, decreasing key intermediates including pyruvate, succinate and fumarate (Fig. 5C). Conversely, GGCT overexpression elevated TCA cycle intermediates (Fig. 5D). This metabolic perturbation further indicated that elevated ROS levels upon GGCT suppression contribute to TCA cycle dysfunction.

To assess whether GGCT metabolic enzymatic activity regulates changes in the TCA cycle, we predicted the structure of GGCT and constructed a mutant (E98A) in PC3 and DU145 cell lines [20] (Fig. S5E, F). Strikingly, GGCT-mutant cells effectively counteracted this metabolic augmentation, rescuing the levels of key intermediates including pyruvate, succinate, and fumarate (Fig. 5D). Moreover, we found that overexpression of GGCT (E98A) mutant significantly reduced cell proliferation compared with overexpression of wild-type GGCT in PC3 and DU145 cells (Fig. 5G–I). Additionally, GGCT knockdown concomitantly reduced 5- oxoproline (5-OP) levels in MHCC97H cells, consistent with 5-OP being the direct enzymatic product of GGCT activity, and exogenous 5-OP rescued proliferation under Gln-deprived conditions (Fig. S5J, K).

To mechanistically dissect how GGCT remodels TCA cycle flux, we first employed [U-^13^C]glutamine isotope tracing to map the rerouting of Gln metabolic pathways. As a result of overexpression GGCT, not only did the level of glutamate m + 5 decrease, but also the levels of downstream metabolites such as α-KG m + 5, succinate m + 4, fumarate m + 4 and malate m + 4 in the TCA cycle were affected (Fig. 5E). Notably, GGCT overexpression paradoxically enhanced the metabolic flux from Gln to GSH m + 5 (Fig. 5E). Additionally, GGCT mutants reversed this alteration, restoring [U-^13^C]-labeling of TCA metabolites and GSH. These data demonstrate that GGCT enzymatically diverts Gln flux away from the TCA cycle, preferentially shunting Gln toward GSH synthesis to sustain redox homeostasis. Conversely, [U-^13^C]glucose tracing showed GGCT overexpression enhanced Glc-derived carbon flux into the TCA cycle (increased m + 2 labeling of succinate, fumarate, malate), which was reversed by the E98A mutant (Fig. 5F).

Considering that energy metabolism is also able to regulate ROS levels, we supplemented sodium pyruvate (the sodium salt form of pyruvate, a key energy substrate for cellular metabolism) or JX06 (a compound that modulates pyruvate flux into the tricarboxylic acid cycle) in GGCT-knockdown cells, respectively. The results showed that compared with the GGCT-knockdown alone group, neither of the above treatments alleviated intracellular ROS accumulation (Fig. 5G, H), indicating that GGCT does not regulate ROS production through energy metabolism. Meanwhile, ATP detection assays confirmed that the ATP level in GGCT-knockdown cells was significantly reduced and rescued in sodium pyruvate or JX06 treatment groups (Fig. 5I). These results illustrate that GGCT regulates intracellular ROS levels primarily via GSH rather than energy metabolism.

Collectively, GGCT functions as a metabolic rheostat that orchestrates mitochondrial homeostasis through diverting Gln flux toward glutathione biosynthesis to fortify redox defense, while enhancing Glc-fueled TCA cycle anaplerosis to sustain cell proliferation.

### Depletion of GGCT inhibits tumor growth in vivo

**Fig. 6:**
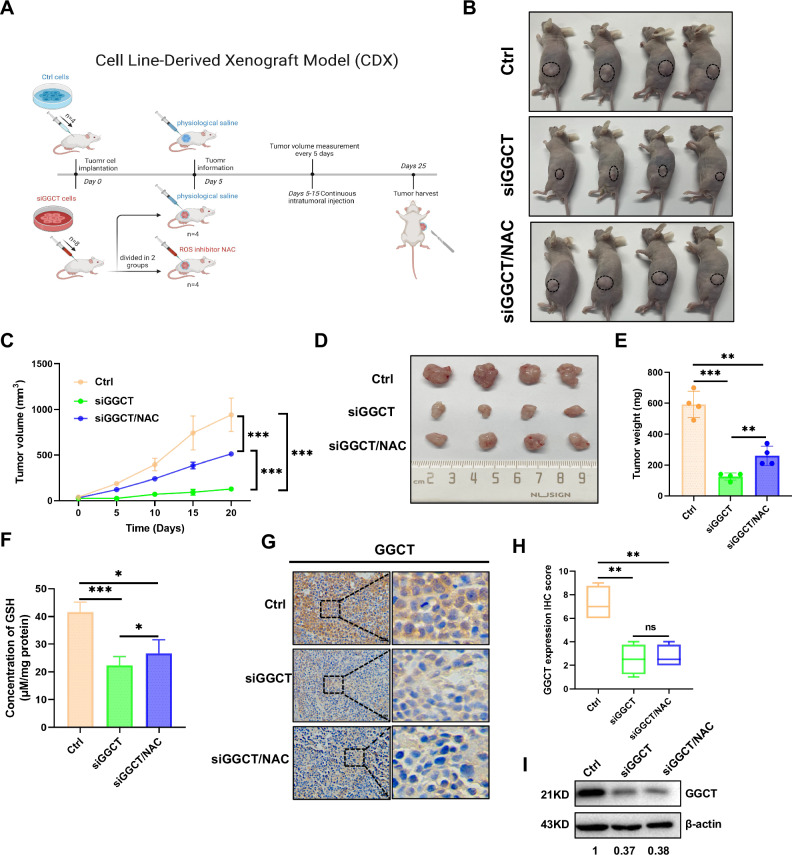
NAC partially rescued GGCT knockdown-mediated tumor suppression. **A** Schematic of the experimental setup for the CDX model. MHCC97H cells (Ctrl, siGGCT) were injected subcutaneously into immunodeficient mice, and mice were divided into different groups: the control group (Ctrl), the siGGCT group treated with siRNA targeting GGCT along with physiological saline, and the siGGCT/NAC group treated with siRNA targeting GGCT along with the ROS inhibitor NAC. Tumor growth was monitored starting from Day 5 to Day 25. **B** Representative images of tumors after 25 days of transplantation from each treatment group: Ctrl (untreated), siGGCT (GGCT knockdown), and siGGCT/NAC (GGCT knockdown with NAC treatment). **C** Tumor volume measurement over time, showing significantly reduced tumor growth in the siGGCT group compared to the Ctrl group. NAC treatment in the siGGCT/NAC group partially restores tumor growth. **D** Images of excised tumors from each group at the end of the study. **E** Quantification of tumor weight for each group, demonstrating a significant decrease in tumor weight in the siGGCT group, with partial recovery in the siGGCT/NAC group. **F** Measurement of oxidative stress levels (GSH concentration) in tumor tissues from each group, indicating elevated oxidative stress in the siGGCT group. NAC treatment in the siGGCT/NAC group reduces oxidative stress levels to near-control levels. **G** Representative immunohistochemical (IHC) staining images of GGCT in xenograft tumor developed from MHCC97H treated as indicated. Scale bar: 20 µm. **H** Quantification of GGCT-positive areas from IHC analysis. **I** Western blot analysis of GGCT protein levels in tumor tissues from each group. (Data represent means ± SD. ns: not significant, *p < 0.05, **p < 0.01, and ***p < 0.001).

To assess the role of GGCT in HCC progression, nude mice were implanted with MHCC97H cells transfected with siGGCT or control siRNA (Ctrl). After tumor development, the siGGCT group received peritumoral injections of NAC or physiological saline, and the tumors were weighed every five days until the samples were harvested for final analysis (Fig. 6A). Consistent with the results of GGCT knockdown in vitro, depletion of GGCT significantly reduced tumor volume and weight compared with control groups. Treatment with NAC rescued tumor growth, indicating that NAC could reverse tumor inhibition induced by GGCT depletion (Fig. 6B–E). Additionally, GSH levels in tumor tissues decreased in the GGCT knockdown group but were restored by NAC, indicating that NAC could elevate the level of GSH by scavenging ROS (Fig. 6F). IHC staining and western blotting confirmed reduced GGCT expression in siGGCT group (Fig. 6G-I). These results suggest that GGCT knockdown can significantly inhibit tumor growth and reduce GSH production and that the addition of the ROS inhibitor NAC can significantly rescue this effect.

## Discussion

As the most abundant amino acid in circulation, Gln exerts an antioxidant defense function against oxidative stress by supporting GSH synthesis [21–23]. Excessive ROS damages nucleic acids, proteins, and lipids to induce cell death, and sufficient intracellular GSH is critical for ROS clearance and cellular homeostasis [24–30]. Our study demonstrated that Gln deprivation severely inhibited the tumor growth of HCC and PCa both in vivo and in vitro, and this occurs partially through reduced GSH levels, resulting in ROS accumulation. GGCT, a key enzyme in tumor metabolism, has been reported to be upregulated in several cancers, and its depletion can exert anticancer effects. However, most studies have focused solely on its involvement in signaling pathway regulation, neglecting its association with Gln metabolism, and even failing to elucidate its mediating role in Gln-modulated redox homeostasis in tumor cells. Prior evidence has indicated that GGCT plays a critical role in maintaining redox balance and prolonging lifespan in red blood cells via the regulation of GSH metabolism, providing a key clue for exploring the crosstalk between Gln and GGCT in cancer [31]. Our study found that GGCT expression was upregulated in a dose-dependent manner with increasing Gln concentrations. Furthermore, GGCT deletion significantly induced ROS production and inhibited GSH levels. In summary, GGCT may mediate the association between Gln metabolism and redox homeostasis in tumor cells, which may fill the mechanistic gap in Gln metabolism-tumor redox regulation.

Protein expression is regulated at multiple levels, including post-transcriptional and post-translational levels [32–35]. Our experimental results demonstrated that the regulation of GGCT expression by Gln occurs neither at the transcriptional level nor via translational regulation, but is achieved at the post-transcriptional level by modulating the stability of GGCT mRNA. MicroRNAs (miRNAs) complement and bind to target mRNAs, leading to mRNA degradation or translation inhibition. A previous study used the TargetScan database indicated miR-205-5p combined with GGCT, which inhibited the growth and metastasis of papillary thyroid cancer [36]. To improve the accuracy of the prediction, we used three prediction tools and discovered nine miRNAs targeting GGCT, further confirming that miR-29b-3p regulates the expression of GGCT in combination with the expression of HCC and PCa tissues in the TCGA database. MiR-29b-3p has been reported to act as a tumor-suppressive miRNA in multiple cancer types, primarily by targeting cancer-associated genes to modulate cell proliferation, apoptosis, invasion, and metastasis [37, 38]. Our study verified that miR-29b-3p directly binds to GGCT mRNA to mediate its expression. To clarify the upstream regulatory mechanism of miR-29b-3p under Gln deprivation conditions, we focused on c-Myc as a key candidate transcription factor. As a highly conserved proto-oncogene-encoded protein, c-Myc acts as a master regulator of cell cycle progression, proliferation, and metabolic reprogramming [39–41]. More importantly, accumulating evidence has established that c-Myc can modulate miRNA transcription by directly binding to their promoter regions [42–47]. providing a critical theoretical basis for investigating its regulatory relationship with miR-29b-3p. Our ChIP assays provided direct mechanistic evidence that Gln deprivation significantly impairs the binding of c-Myc to the miR-29b-3p promoter. Further functional validation experiments clarified the regulatory logic between these two molecules. Specifically, under Gln+ conditions, highly active c-Myc represses miR-29b-3p transcription, leading to a substantial reduction in its expression. In contrast, Gln deprivation abrogates c-Myc-mediated transcriptional repression, resulting in a significant upregulation of miR-29b-3p expression. Notably, this effect can be reversed by c-Myc overexpression. Taken together, our study refines the critical Gln/c-Myc/miR-29b-3p/GGCT regulatory axis, clearly illustrating how Gln modulates GGCT expression.

As a core energy and biosynthetic substrate for tumor cells, Gln metabolic reprogramming has emerged as a research hotspot in tumor metabolism in recent years. Gln is catalyzed by glutaminase to generate α-ketoglutarate (α-KG), which can directly feed into the TCA cycle. Accumulating evidence has verified that perturbation of Gln metabolism can significantly affect TCA cycle flux and tumor cell proliferation [48–50]. For instance, Hu et al. demonstrated that Gln deprivation reduces the levels of key TCA cycle intermediates, thereby inhibiting tumor cell proliferation [51]. Shen et al. also reported that aberrant Gln metabolism is closely associated with the malignant phenotype of tumor cells [52]. Yet, the interaction mechanism between glutamine (Gln) and glucose remains ambiguous. Notably, our metabolomic results demonstrated a close correlation between GGCT expression and Gln metabolic flux in tumor cells. Further isotope labeling experiments confirmed that upon GGCT overexpression, Gln is preferentially shunted to the GSH synthesis pathway, with a significantly reduced proportion entering the TCA cycle. This indicates the existence of a GGCT-mediated Gln metabolic shunt in tumor cells: Gln is prioritized for conversion to GSH, which scavenges ROS, alleviates oxidative stress, and ultimately promotes tumor cell proliferation. Then, how do tumor cells sustain rapid proliferation when a large portion of glutamine is shunted toward GSH synthesis via GGCT-mediated regulation, consequently reducing glutamine flux into TCA cycle? Using isotope-labeled glucose metabolomics assays, we found that GGCT overexpression significantly promotes glucose influx into the TCA cycle to sustain cell proliferation. Although energy metabolism status can also regulate intracellular ROS levels (e.g., glucose deprivation leads to a marked increase in cellular ROS) [53–56], our study demonstrated that supplementation with energy substrates such as sodium pyruvate in GGCT-knockdown cell models did not induce significant alterations in intracellular ROS levels. This finding provides compelling evidence that GGCT reduces ROS primarily by regulating Gln shunting to GSH, rather than through modulating energy metabolism pathways to exert its pro-proliferative effects. Collectively, GGCT emerges as a pivotal metabolic orchestrator in HCC and PCa, functioning as a bidirectional switch that diverts Gln toward GSH biosynthesis for redox defense while enhancing Glc-fueled TCA anaplerosis to sustain cell proliferation.

Collectively, our findings demonstrate that GGCT can coordinately regulate both Gln and Glc metabolism and drives cell proliferation via two distinct mechanisms. On the one hand, GGCT targets the Gln metabolic pathway to promote the conversion of GSH, thereby scavenging ROS and relieving oxidative stress constraints on cell proliferation. On the other hand, GGCT overexpression enhances the metabolic flux of Glc into TCA cycle, providing sufficient energy and critical metabolic intermediates required for rapid cell proliferation.

## Supplementary information

supplementary figure legends

FigS1

FigS2

FigS3

FigS4

FigS5

TableS1

TableS2

TableS3

TableS4

wb raw data

## Data Availability

The data used is confidential.
