# Supplementary material for: Metabolic orchestration driven by GGCT: diverting glutamine to glutathione biosynthesis while enhancing glucose anaplerosis for tumor proliferation
Source: Cell Death Dis. 2026 Mar 24;17(1):358. doi: 10.1038/s41419-026-08619-y (PMC13039682; doi:10.1038/s41419-026-08619-y)
Supplement: Supplementary file 1 — supplementary figure legends [file 41419_2026_8619_MOESM1_ESM.doc]

**Supplementary Figure legends**

Fig. S1 Gln promoted proliferation, affected ROS and GSH levels in vitro and in vivo. (A) Volcano plot from dataset GSE14086 showing differentially expressed genes between control and glutamine- treated groups. Red and blue dots represent significantly upregulated and downregulated genes, respectively. (B) KEGG and GO enrichment analyses of differentially expressed genes. The left panel displays KEGG pathways and the right panel shows GO biological processes. (C) Cell proliferation assays of LNCaP and C4-2 cells treated with 0 and 4 mM Gln. (D-E) Colony formation assays for LNCaP and C4-2 cells treated with 0 and 4 mM Gln. (F) The western blot analysis was applied to detect the protein level of CCNB1, CDK1 and p-CDK1 in LNCaP and C4-2 cells treated with 0mM and 4mM Gln. (G) Tumor weights of subcutaneous xenograft tumor model were measured after removal. (H) Tumor growth curves of subcutaneous xenograft tumor model developed from LNCaP cells treatment with indication (n = 3). (I) The GSH levels of LNCaP and C4-2 cells treated with 0 mM and 4 mM Gln with or without N- acetylcysteine (NAC) as an antioxidant. (J) Representative images of DCFH-DA staining for ROS detection in LNCaP and C4-2 cells under different Gln conditions, with or without NAC treatment. Scale bar:100 µm. (K) Relative fluorescence of DCFH-DA staining for ROS detection in LNCaP and C4-2 cells under different Gln conditions, with or without NAC treatment in flowcytometer. (L) The cell viability of LNCaP and C4-2 cells treated with 0 mM and 4 mM Gln, with and without NAC. (Data represent means ± SD. ns: not significant, **p*<0.05, ***p* < 0.01, and ****p* < 0.001).

Fig. S2 Expression of related genes of GSH metabolism and the expression of GGCT in various cancers. (A) Box plots displaying the expression levels of genes involved in the GSH metabolism (GCLC, GCLM) and GGT family members (GGT1, GGT2, GGT3, GGT4, GGT5, GGT6, and GGT7) in tumor versus normal tissues based on TCGA datasets. Red and blue boxes represent tumor and normal tissue samples, respectively. (B) Box plot showing GGCT expression across various cancer types in TCGA datasets, comparing tumor (red) and normal (blue) samples. Statistical significance was determined **p* < 0.05, ** *p* < 0.01, and *** *p* < 0.001.

Fig. S3 Expression of miR-29b-3p in HCC and PCa and its impact on proliferation. (A) Western blot analysis was performed to detect GGCT protein levels in MHCC97H and LNCaP cells under Gln deprivation (0mM) and supplementation (4mM) conditions. Cells were treated with DMSO or various proteasome inhibitors (MG132, CQ) to assess GGCT stability. β-actin serves as a loading control. (B) Relative activities of GGCT promoter under Gln+ and Gln- conditions were detected by Dual luciferase system in colon cancer MHCC97H. (C) Paired dot plots comparing expression levels of candidate miRNAs in HCC (top row) and PCa (bottom row) tumor tissues versus adjacent normal tissues. (****p* < 0.001, ***p* < 0.01). (D) **Correlation analysis between miR-29b-3p/ miR-769-5p and GGCT expression** based on TCGA datanbase. (E) qRT-PCR detected the expression of GGCT mRNA and miR-29b-3p in 5 pairs of HCC tissues. (F) qRT-PCR detected the expression of miR- 29b-3p in MHCC97H and LNCaP cells, with NC, miR-29b-3p mimics or inhibitor transfection. (G) CCK8 assay detected the cell proliferation ability of MHCC97H and LNCaP cells transfected with NC, miR-29b-3p mimics and inhibitor. (Data represent means ± SD. ns: not significant, **p*<0.05, ***p* < 0.01, and ****p* < 0.001).

Fig. S4 GGCT affects the proliferation by disrupting the redox balance in PCa cells. (A) KEGG pathway enrichment analysis for differentially expressed genes in HCC and PCa cells upon GGCT knockdown. (B) GO enrichment analysis for HCC and PCa cells, showing biological processes affected by GGCT knockdown, particularly those involved in cell cycle regulation, proliferation, and response to oxidative stress. (C) Cell proliferation assays for LNCaP and C4-2 following GGCT knockdown, and PC3 cells treated with GGCT under the conditions of Gln deprivation. (D) Colony formation assays in LNCaP and C4-2 knocking down GGCT cells and PC3 treated with GGCT overwxpression under Gln deprivation. (E) Analysis of cell cycle distribution via flow cytometry in LNCaP and C4-2 cells as indicated treatment. (F) Western blot analysis of cell cycle-related proteins (CCNB1, CDK1 and p-CDK1 ) in LNCaP and C4-2 cells following GGCT knockdown. (G) Quantification of GSH levels in LNCaP and C4-2 cells under different treatment conditions (Ctrl, siGGCT, siGGCT/NACl). (H) Representative images of DCFH-DA staining in LNCaP and C4-2 cells to assess ROS levels after GGCT knockdown, with and without NAC treatment. Scale bar:100 µm. (J-K) Quantification of DCFH-DA fluorescence intensity as indicated treatment. (I) Quantitative analysis of cell viability in LNCaP and C4-2 cells following GGCT knockdown and pretreatment of NAC. (L) Quantification of cell viability in PC3 cells with GGCT overexpression, under varying Gln concentrations (0 mM and 4 mM Gln). (M) Representative DCFH-DA staining images in PC3 cells overexpressing GGCT and treated with 0 mM and 4 mM Gln. Scale bar:100 µm. (N) The proliferation ability of MHCC97H and LNCaP cells (NC and miR-29b-3p mimics and miR- 29b-3p mimics/PCMV-Flag-GGCT), as measured by Cell Counting Kit-8 assay. (Data represent means ± SD. ns: not significant, **p*<0.05, ***p* < 0.01, and ****p* < 0.001).

Fig. S5 The impact of GGCT on mitochondrial and the function of GGCT mutation on cell proliferation. (A-B) Transmission electron microscopy images of mitochondrial in HepG2 and C4-2 cells with treatment as indicated (siNC, control siNC expression vector. siGGCT, siRNA expression vector against GGCT). Arrows indicate elongated mitochondria. Red arrows indicate mitochondria with altered morphology in GGCT knockdown cells. Scale bar: 2 μm. (C-D) Detection of oxygen consumption rate (OCR) in GGCT-knockdown cells. Basal glycolysis capacity, maximal glycolysis capacity, reserved glycolysis capacity in siNC and siGGCT cells. (E) Structural model of GGCT showing the wild-type (E98) and mutant (E98A) forms. The mutation site was highlighted, indicating the substitution of glutamic acid (E) with alanine (A) at position 98. (F) Western blot analysis of GGCT expression in PC3 and DU145 cells transfected with eGFP, GGCT-eGFP, or GGCT-E98A-eGFP constructs. GGCT expression is confirmed in cells expressing the wild-type and mutant GGCT constructs. β-actin serves as a loading control. (G) Cell proliferation assays for PC3 and DU145 cells transfected with eGFP, GGCT-eGFP, or GGCT-E98A-eGFP. (H) Colony formation assays in PC3 cells with eGFP, GGCT-eGFP, or GGCT-E98A-eGFP constructs. (I) Colony formation assays in DU145 cells with eGFP, GGCT-eGFP, or GGCT-E98A-eGFP constructs. (J) Typical extract ion chromatographies of 5-Oxoproline in metabolic target analysis of MHCC97H treated with siGGCT RNA and relative intensity of 5-Oxoproline (n=3). (K) The cell viability of MHCC97H and LNCaP treated with 5-Oxoproline under Gln deprivation conditions. (Data represent means ± SD. ns: not significant, **p*<0.05, ***p* < 0.01, and ****p* < 0.001).
