# Supplementary figures and images for: Metabolic orchestration driven by GGCT: diverting glutamine to glutathione biosynthesis while enhancing glucose anaplerosis for tumor proliferation
Source: Cell Death Dis. 2026 Mar 24;17(1):358. doi: 10.1038/s41419-026-08619-y (PMC13039682; doi:10.1038/s41419-026-08619-y)

**A**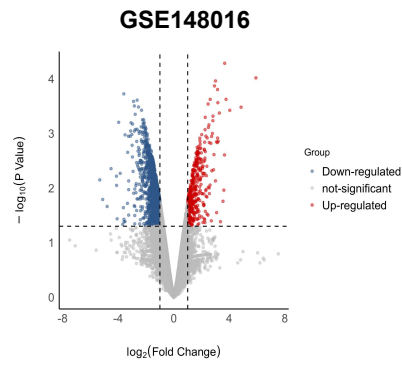**B**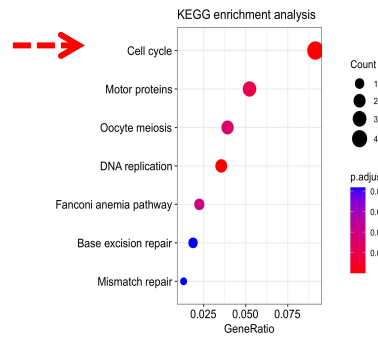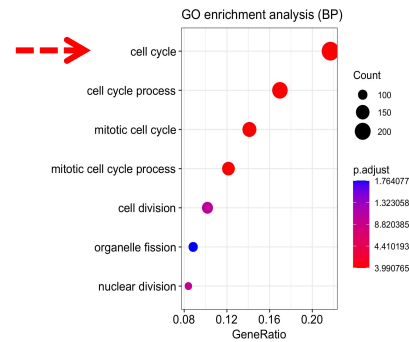**C**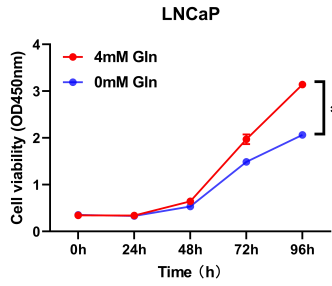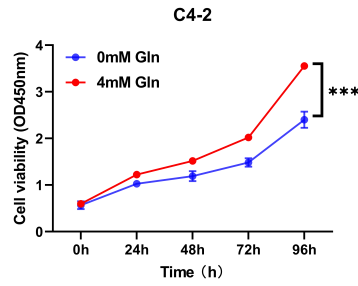**D**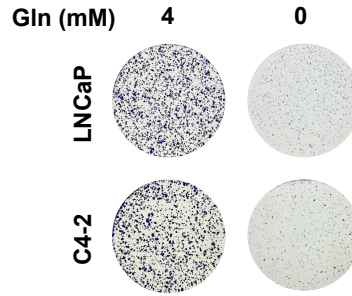**E**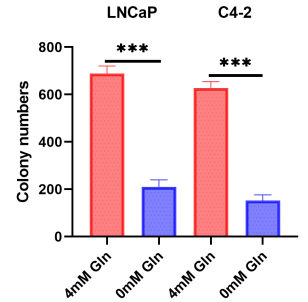**F**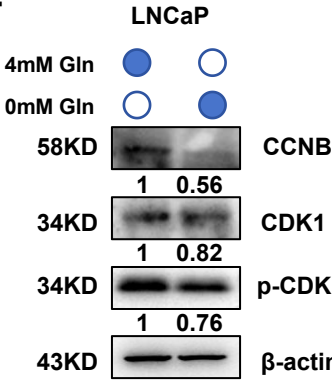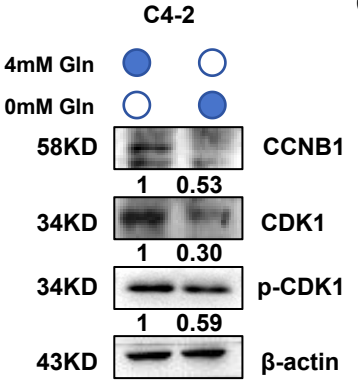**G**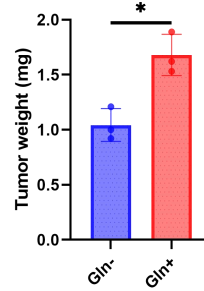**H**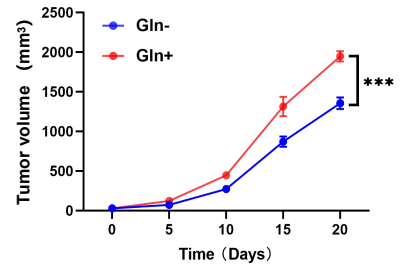**I**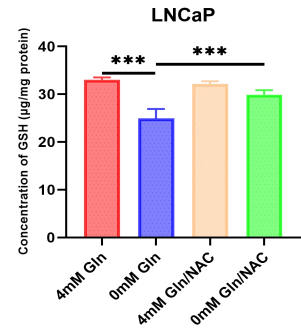**J**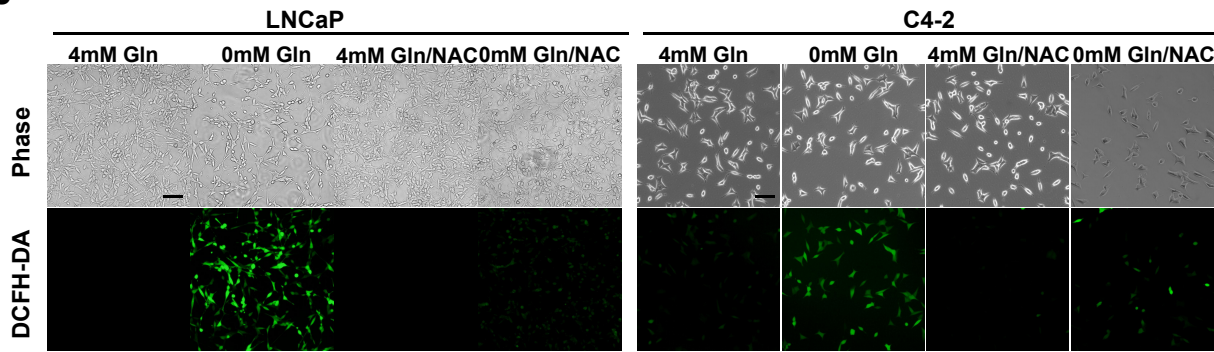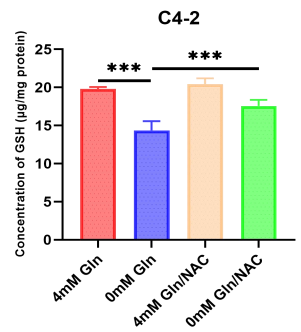**K**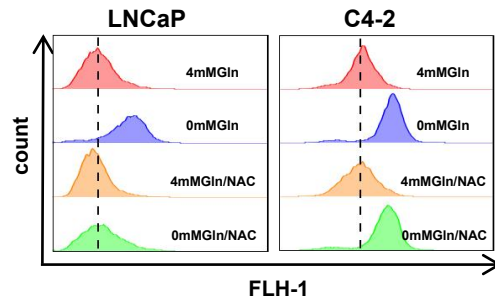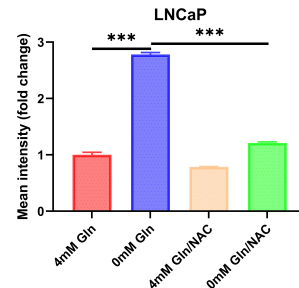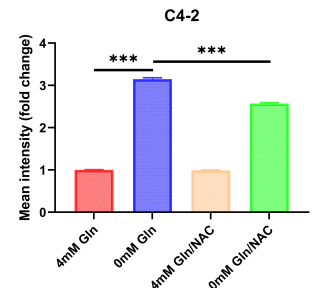**L**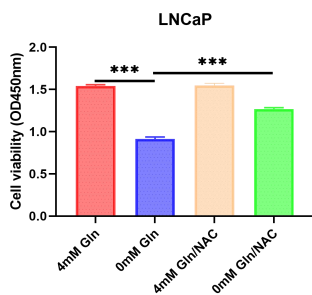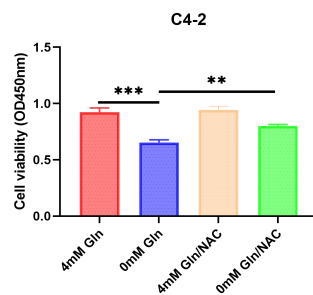

Supplement: Supplementary file 2 — FigS1 [file 41419_2026_8619_MOESM2_ESM.pdf]

**A**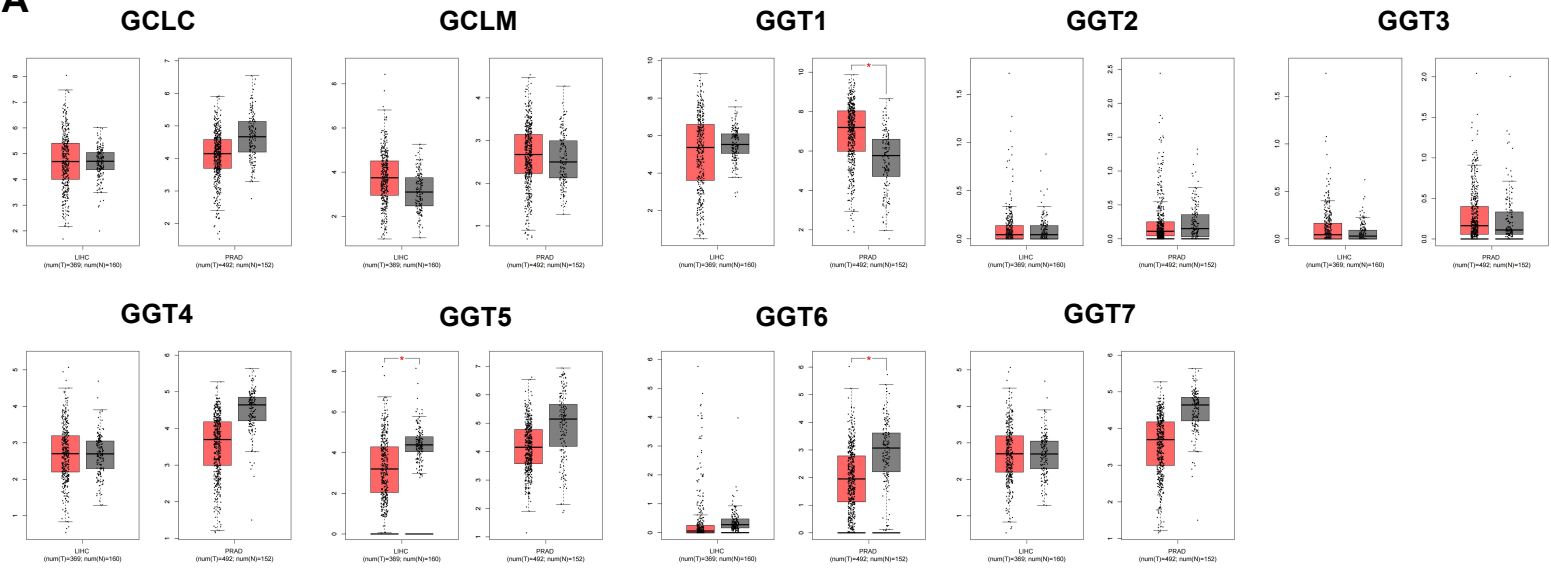**B**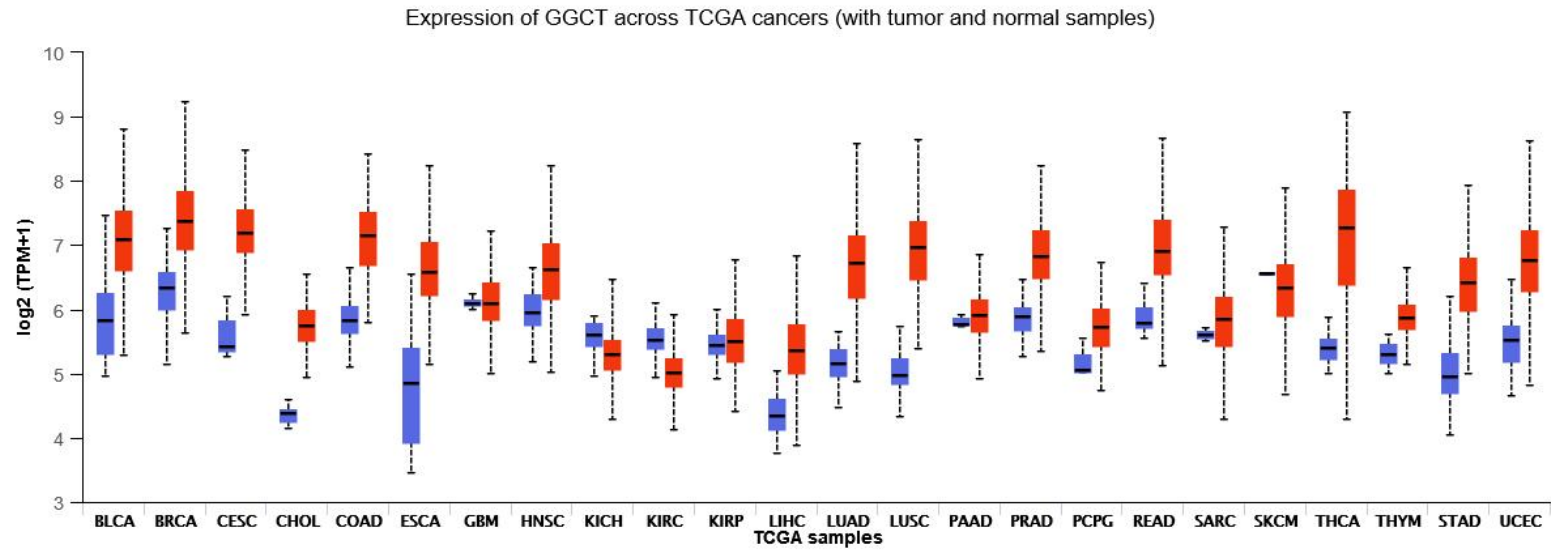

Supplement: Supplementary file 3 — FigS2 [file 41419_2026_8619_MOESM3_ESM.pdf]

**A**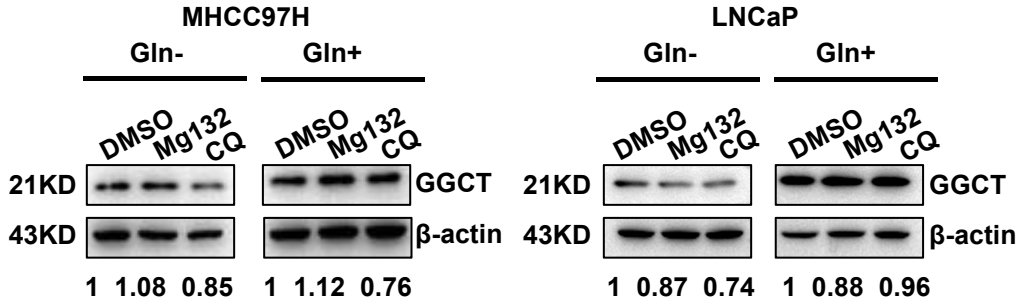**B**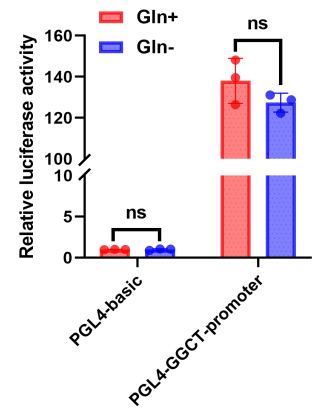**C**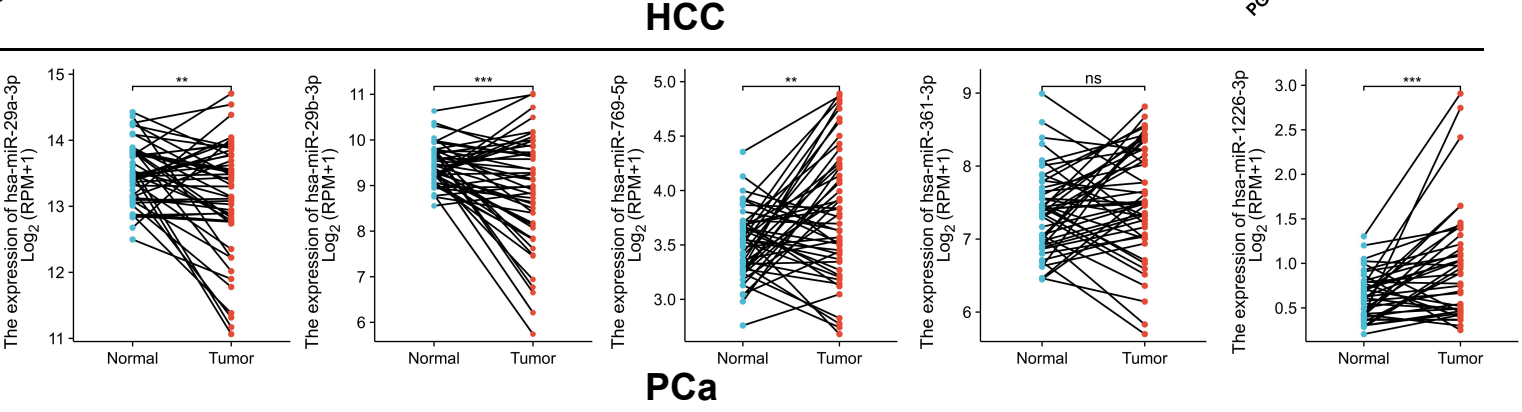**D**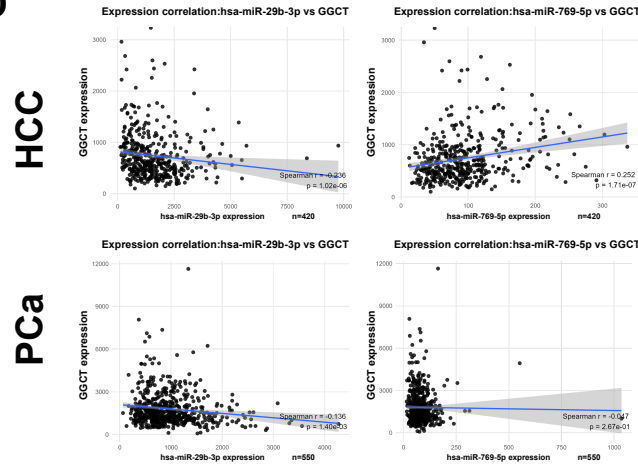**E**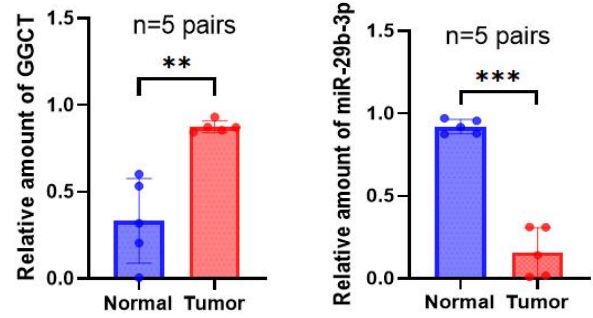**F**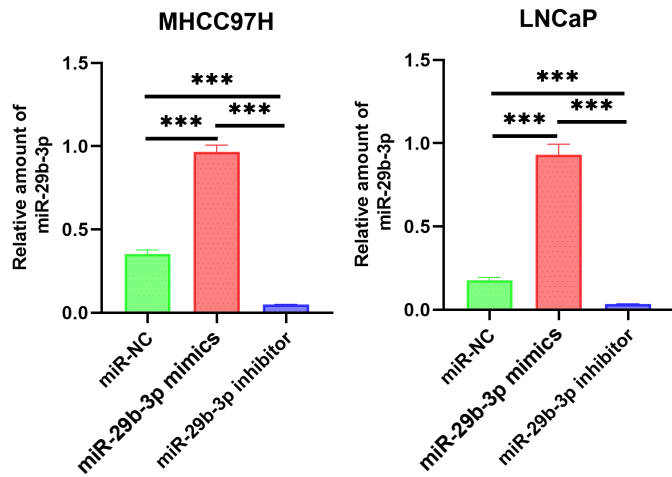**G**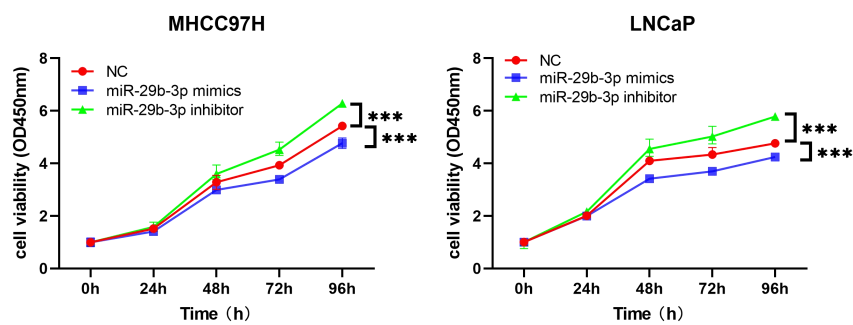

Supplement: Supplementary file 4 — FigS3 [file 41419_2026_8619_MOESM4_ESM.pdf]

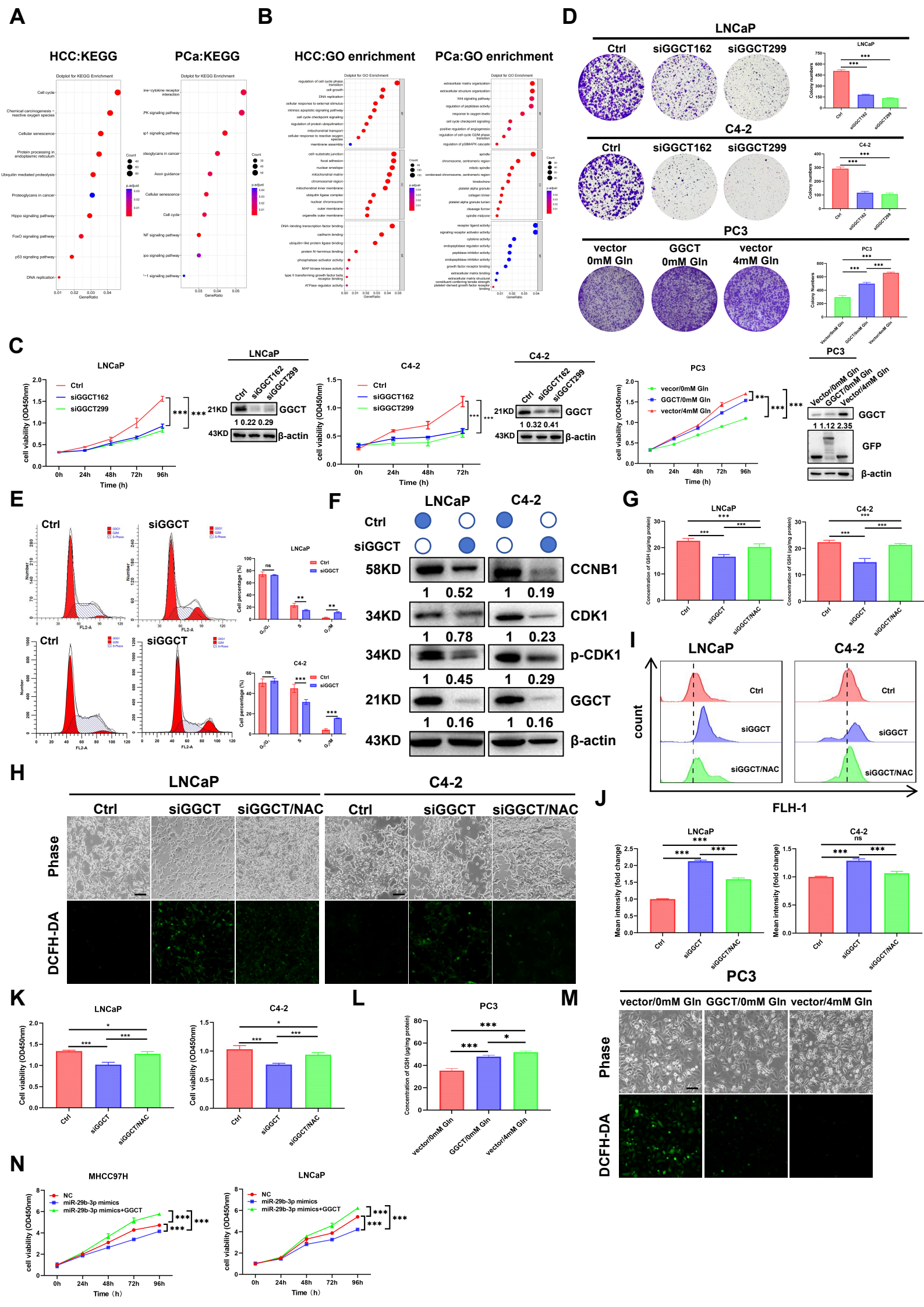

Supplement: Supplementary file 5 — FigS4 [file 41419_2026_8619_MOESM5_ESM.pdf]

**A****HepG2**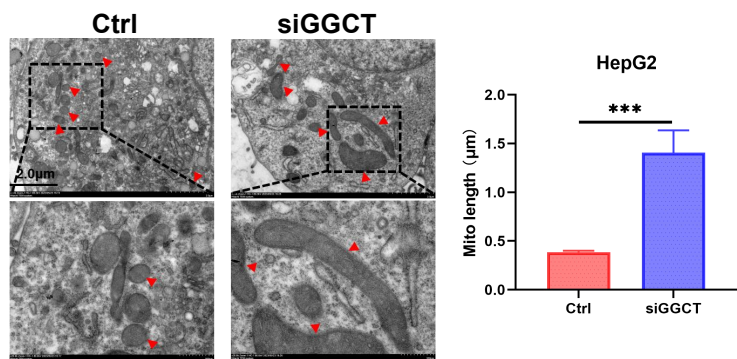**B****C4-2**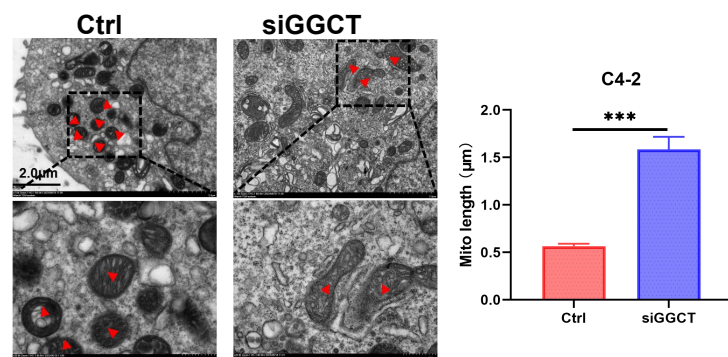**C**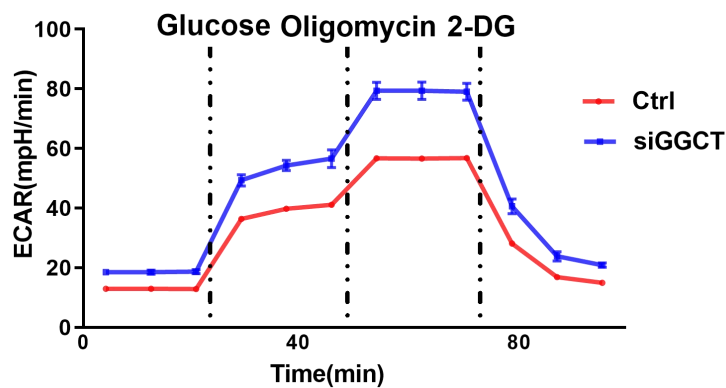**D**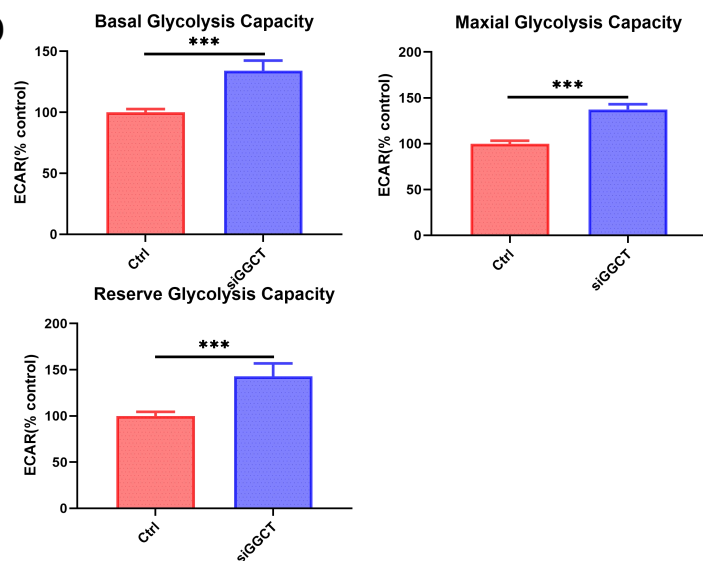**E**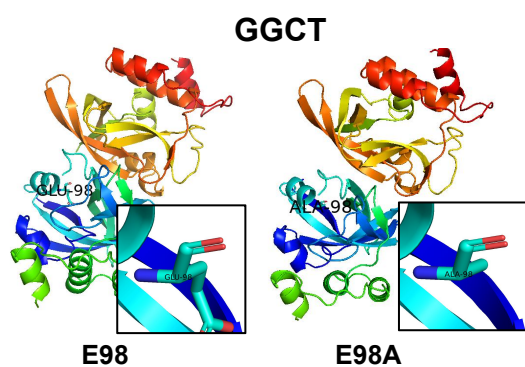**F**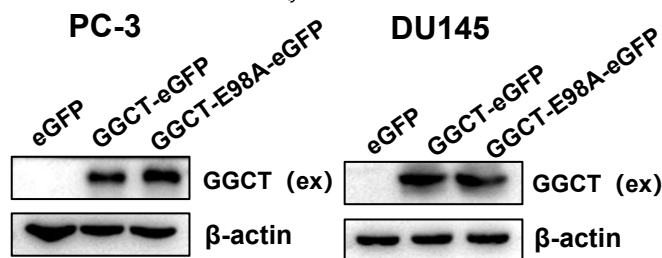**G**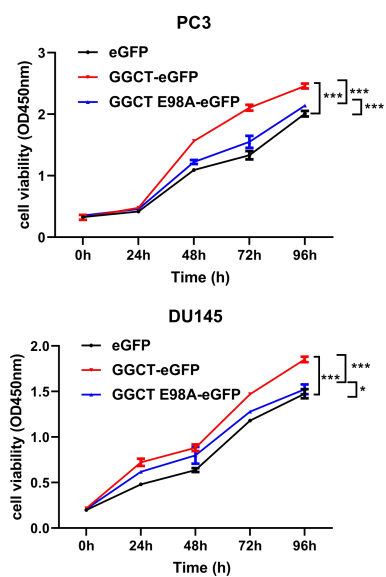**H**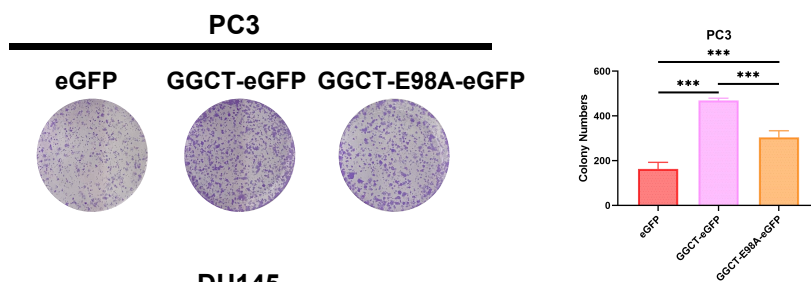**I**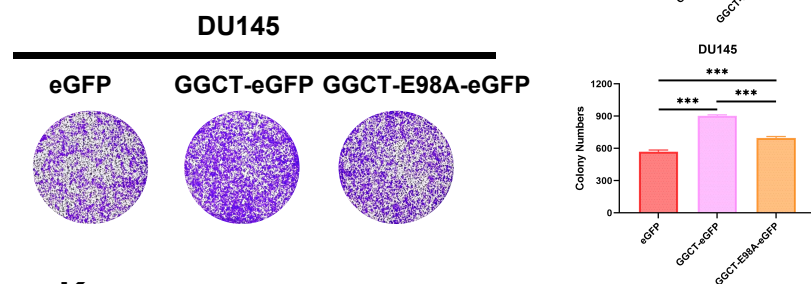**J**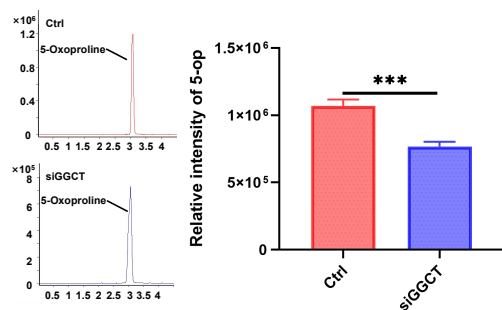**K**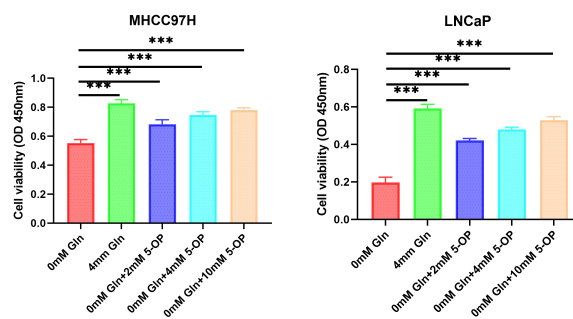

Supplement: Supplementary file 6 — FigS5 [file 41419_2026_8619_MOESM6_ESM.pdf]
