# Supplementary material for: Metabolic orchestration driven by GGCT: diverting glutamine to glutathione biosynthesis while enhancing glucose anaplerosis for tumor proliferation
Source: Cell Death Dis. 2026 Mar 24;17(1):358. doi: 10.1038/s41419-026-08619-y (PMC13039682; doi:10.1038/s41419-026-08619-y)
Supplement: Supplementary file 7 — TableS1 [file 41419_2026_8619_MOESM7_ESM.doc]

**Table S1 siRNA target sequences**

| Target | Primers | Sequences (5′→3′) |
| --- | --- | --- |
| siGGCT162 | Forward | GGAGUAUCAAGAGAAGUUATT |
|  | Reverse | UAACUUCUCUUGAUACUCCTT |
| siGGCT299 | Forward | UGGAGUAUCAAGAGAAGUUTT |
|  | Reverse | AACUUCUCUUGAUACUCCATT |
| siNC | Forward | UUCUCCGAACGUGUCACGUTT |
|  | Reverse | ACGUGACACGUUCGGAGAATT |
