# Supplementary material for: Metabolic orchestration driven by GGCT: diverting glutamine to glutathione biosynthesis while enhancing glucose anaplerosis for tumor proliferation
Source: Cell Death Dis. 2026 Mar 24;17(1):358. doi: 10.1038/s41419-026-08619-y (PMC13039682; doi:10.1038/s41419-026-08619-y)
Supplement: Supplementary file 8 — TableS2 [file 41419_2026_8619_MOESM8_ESM.doc]

**Table S2 Primers of human genes used for the qPCR reaction**

| Target | Species | Primers | Sequences (5′→3′) |
| --- | --- | --- | --- |
| β-Actin | Human | Forward | CATGTACGTTGCTATCCAGGC |
|  |  | Reverse | CTCCTTAATGTCACGCACGAT |
| GGCT | Human | Forward | TGGCAATTCCCAAGGCAAAAC |
|  |  | Reverse | CCCCTTCTTGCTCATCCAGAG |
| S1 | Human | Forward | GCACCTCCTGACTGATTAG |
|  |  | Reverse | TTTCTCCGTCTGACTTTCC |
| S2 | Human | Forward | TGCCTCTGACTTCTCCTG |
|  |  | Reverse | GTTGCCCAGACAAAGGTT |
