# Supplementary material for: Metabolic orchestration driven by GGCT: diverting glutamine to glutathione biosynthesis while enhancing glucose anaplerosis for tumor proliferation
Source: Cell Death Dis. 2026 Mar 24;17(1):358. doi: 10.1038/s41419-026-08619-y (PMC13039682; doi:10.1038/s41419-026-08619-y)
Supplement: Supplementary file 9 — TableS3 [file 41419_2026_8619_MOESM9_ESM.doc]

**Table S3**  Antibodies used in this study

| Antibodies | Source | Identifier |
| --- | --- | --- |
| GGCT | Abcam | ab198503 |
| GGCT | Proteintech | 16257-1-AP |
| CCNB1 | Proteintech | 55004-1-AP |
| CDK1 | Proteintech | 19532-1-AP |
| β-actin | ABclonal | AC026 |
| c-Myc | Proteintech | 10828-1-AP |
| p-CDK1 | ABclonal | AP1466 |
