# Supplementary material for: Metabolic orchestration driven by GGCT: diverting glutamine to glutathione biosynthesis while enhancing glucose anaplerosis for tumor proliferation
Source: Cell Death Dis. 2026 Mar 24;17(1):358. doi: 10.1038/s41419-026-08619-y (PMC13039682; doi:10.1038/s41419-026-08619-y)
Supplement: Supplementary file 10 — TableS4 [file 41419_2026_8619_MOESM10_ESM.doc]

**Table S4 miRNA target sequences**

| Target | Primers | Sequences (5′→3′) |
| --- | --- | --- |
| miR-29b-3p mimics | Forward | UAGCACCAUUUGAAAUCAGUGUU |
| miR-29b-3p inhibitor | Forward | AACACUGAUUUCAAAUGGUGCUA |
| Negative control | Forward | UCACAACCUCCUAGAAAGAGUAGA |
|  | Reverse | UCUACUCUUUCUAGGAGGUUGUGA |
