# Supplementary material for: Metabolic orchestration driven by GGCT: diverting glutamine to glutathione biosynthesis while enhancing glucose anaplerosis for tumor proliferation
Source: Cell Death Dis. 2026 Mar 24;17(1):358. doi: 10.1038/s41419-026-08619-y (PMC13039682; doi:10.1038/s41419-026-08619-y)
Supplement: Supplementary file 11 — wb raw data [file 41419_2026_8619_MOESM11_ESM.pdf]

Fig 1G

M 1 2

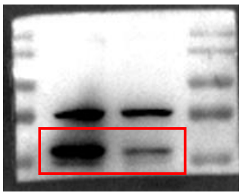

CCNB1 (58KD)

1 lane: MHCC97H 4mM Gln

2 lane: MHCC97H 0mM Gln

M 1 2

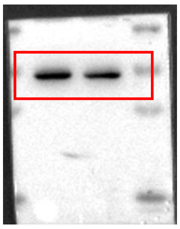

CDK1 (34KD)

1 lane: MHCC97H 4mM Gln

2 lane: MHCC97H 0mM Gln

M 1 2

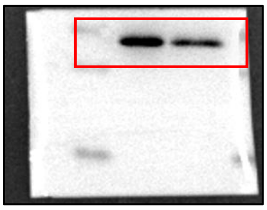

p-CDK1 (34KD)

1 lane: MHCC97H 4mM Gln

2 lane: MHCC97H 0mM Gln

M 1 2

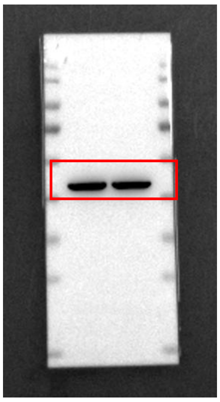

β-actin (43KD)

1 lane: MHCC97H 4mM Gln

2 lane: MHCC97H 0mM Gln

M 1 2

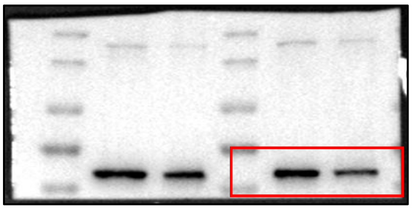

CCNB1 (58KD)

1 lane: HepG2 4mM Gln

2 lane: HepG2 0mM Gln

M 1 2

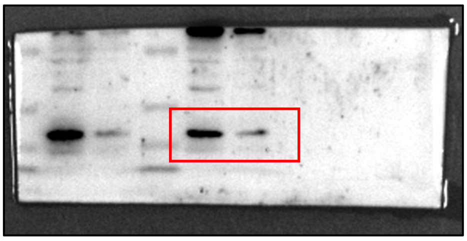

CDK1 (34KD)

1 lane: HepG2 4mM Gln

2 lane: HepG2 0mM Gln

M 1 2

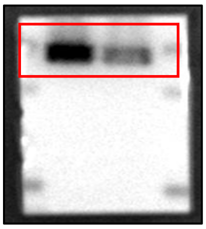

P-CDK1 (34KD)

1 lane: HepG2 4mM Gln

2 lane: HepG2 0mM Gln

M 1 2

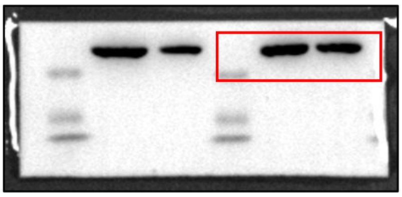

β-actin (43KD)

3 lane: HepG2 4mM Gln

4 lane: HepG2 0mM Gln

Fig 2F

M 1 2 3 4 5 6 7 8 9 10

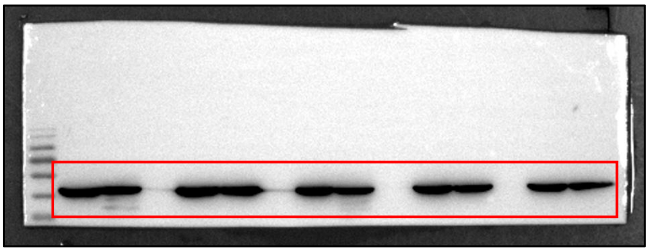

β-actin (43KD)

1lane: N1　2 lane: T1
3lane: N2　4 lane: T2
5lane: N3　6 lane: T3
7lane: N4　8 lane: T4
9lane: N5　10 lane: T5

M 1 2 3 4 5 6 7 8 9 10

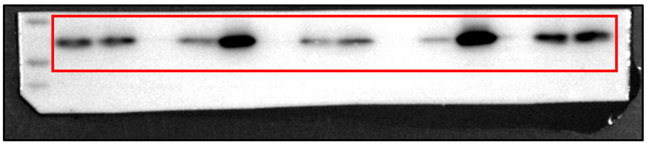

GGCT (21KD)

1lane: N1　2 lane: T1
3lane: N2　4 lane: T2
5lane: N3　6 lane: T3
7lane: N4　8 lane: T4
9lane: N5　10 lane: T5

M 1 2 3 4 5 6 7 8 9 10

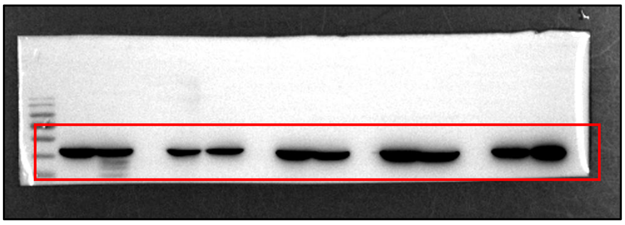

β-actin (43KD)

1lane: N6　2 lane: T6
3lane: N7　4 lane: T7
5lane: N8　6 lane: T8
7lane: N9　8 lane: T9
9lane: N10　10 lane: T10

M 1 2 3 4 5 6 7 8 9 10

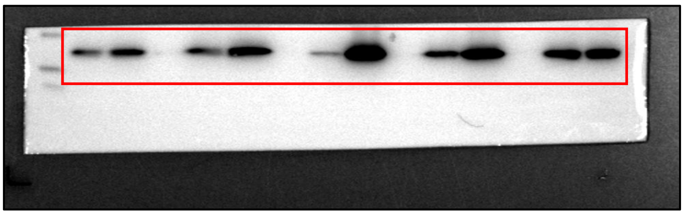

GGCT (21KD)

1lane: N6　2 lane: T6
3lane: N7　4 lane: T7
5lane: N8　6 lane: T8

7lane: N9   8 lane: T9
9lane: N10   10 lane: T10

Fig 2H

M 1 2 3 4 5 6

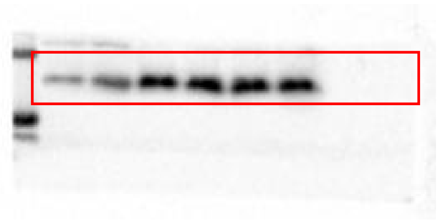

GGCT (21KD)

1lane:HepG2 0mM Gln
2lane:HepG2 0.1mM Gln
3lane:HepG2 0.5mM Gln
4lane:HepG2 1mM Gln
5lane:HepG2 2mM Gln
6lane:HepG2 4mM Gln

M 1 2 3 4 5 6

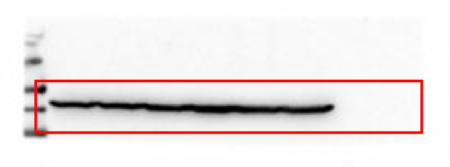

β-actin (43KD)

1lane:HepG2 0mM Gln
2lane:HepG2 0.1mM Gln
3lane:HepG2 0.5mM Gln
4lane:HepG2 1mM Gln
5lane:HepG2 2mM Gln
6lane:HepG2 4mM Gln

M 1 2 3 4 5 6

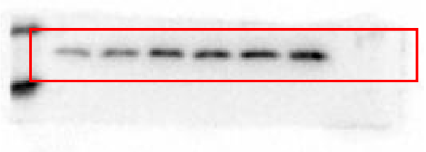

GGCT (21KD)

1lane:MHCC97H 0mM Gln
2lane:MHCC97H 0.1mM Gln
3lane:MHCC97H 0.5mM Gln
4lane:MHCC97H 1mM Gln
5lane:MHCC97H 2mM Gln
6lane:MHCC97H 4mM Gln

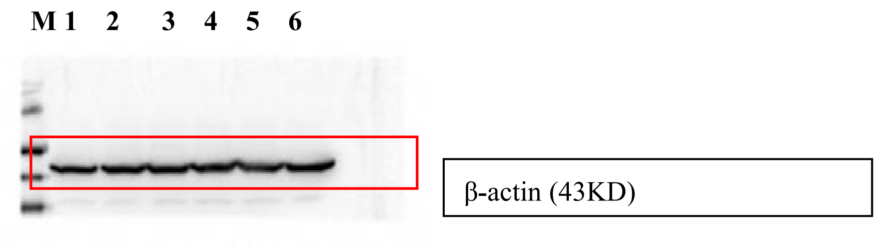

1lane:MHCC97H 0mM Gln
2lane:MHCC97H 0.1mM Gln
3lane:MHCC97H 0.5mM Gln
4lane:MHCC97H 1mM Gln
5lane:MHCC97H 2mM Gln
6lane:MHCC97H 4mM Gln

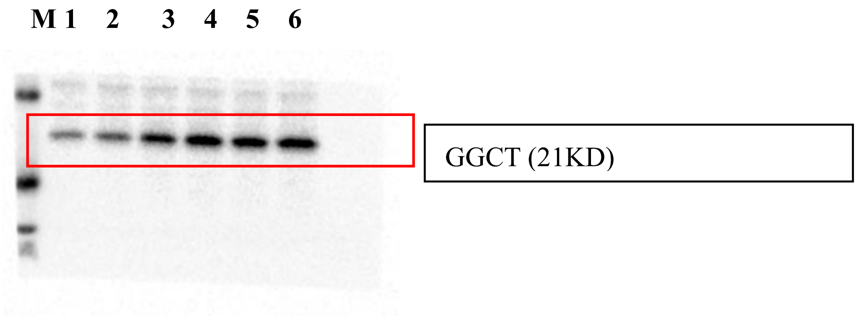

1lane:LNCaP 0mM Gln
2lane:LNCaP 0.1mM Gln
3lane:LNCaP 0.5mM Gln
4lane:LNCaP 1mM Gln
5lane:LNCaP 2mM Gln
6lane:LNCaP 4mM Gln

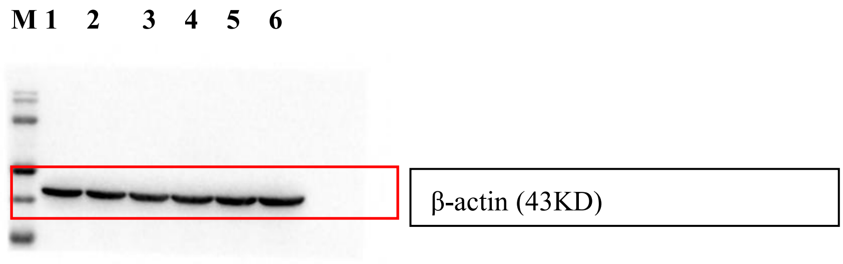

1lane:LNCaP 0mM Gln
2lane:LNCaP 0.1mM Gln
3lane:LNCaP 0.5mM Gln
4lane:LNCaP 1mM Gln
5lane:LNCaP 2mM Gln
6lane:LNCaP 4mM Gln

Fig 3A

M 1 2 3 4 5 6

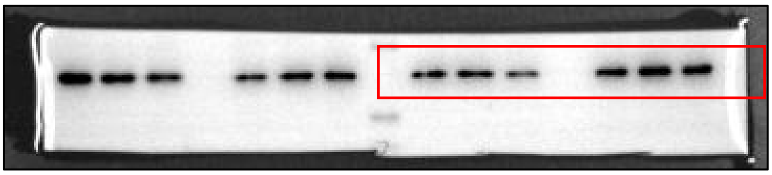

GGCT (21KD)

1lane:MHCC97H Gln-/DMSO

2lane:MHCC97H Gln-/MG132

3lane:MHCC97H Gln-/CQ

4lane:MHCC97H Gln+/DMSO

5lane:MHCC97H Gln+/MG132

6lane:MHCC97H Gln+/CQ

M 1 2 3 4 5 6

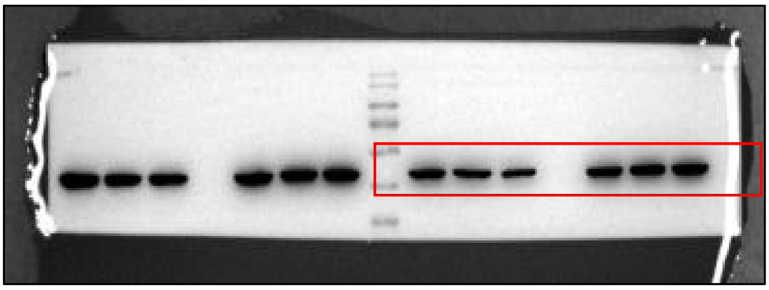

β-actin (43KD)

1lane:MHCC97H Gln-/DMSO

2lane:MHCC97H Gln-/MG132

3lane:MHCC97H Gln-/CQ

4lane:MHCC97H Gln+/DMSO

5lane:MHCC97H Gln+/MG132

6lane:MHCC97H Gln+/CQ

M 1 2 3 4 5 6

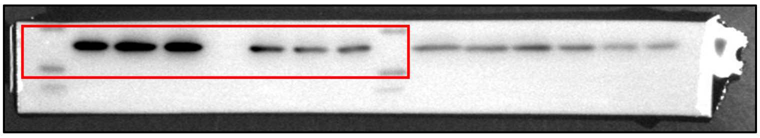

GGCT (21KD)

1lane:LNCaP Gln+/DMSO

2lane:LNCaP Gln+/MG132

3lane:LNCaP Gln+/CQ

4lane:LNCaP Gln-/DMSO

5lane:LNCaP Gln-/MG132

6lane:LNCaP Gln-/CQ

M 1 2 3 4 5 6

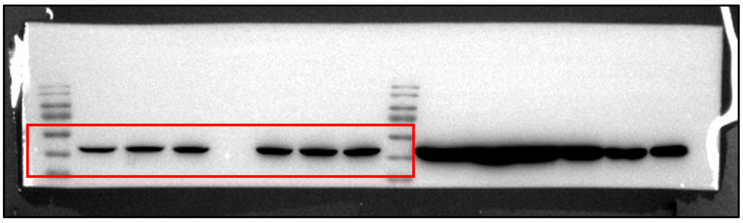

β-actin (43KD)

1lane:LNCaP Gln+/DMSO
2lane:LNCaP Gln+/MG132
3lane:LNCaP Gln+/CQ
4lane:LNCaP Gln-/DMSO
5lane:LNCaP Gln-/MG132
6lane:LNCaP Gln-/CQ

Fig 3F

1 2 3

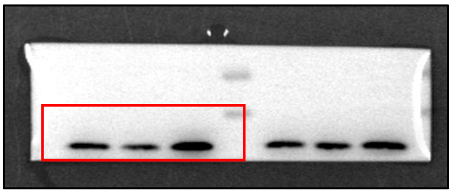

GGCT (21KD)

1lane:MHCC97H miR-NC
2lane:MHCC97H miR-29b-3p mimic
3lane:MHCC97H miR-29b-3p inhibitor

1 2 3

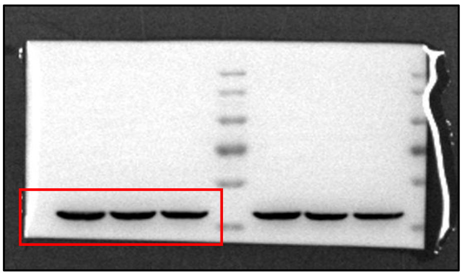

β-actin (43KD)

1lane:MHCC97H miR-NC
2lane:MHCC97H miR-29b-3p mimic
3lane:MHCC97H miR-29b-3p inhibitor

M 1 2 3

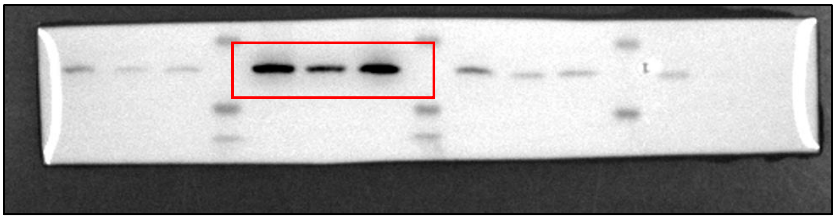

GGCT (21KD)

1lane:LNCaP miR-NC
2lane:LNCaP miR-29b-3p mimic
3lane:LNCaP miR-29b-3p inhibitor

M 1 2 3

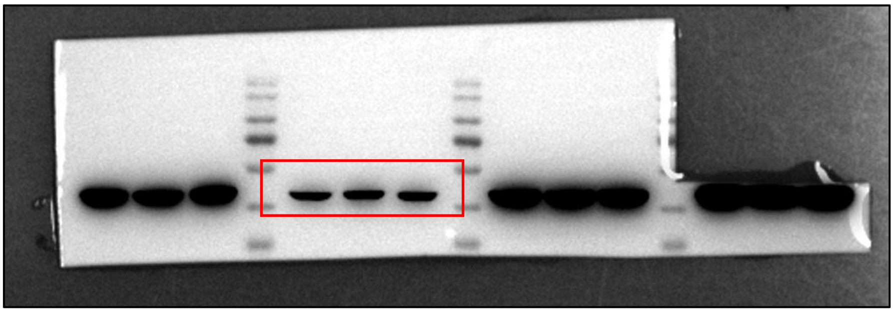

β-actin (43KD)

1lane:LNCaP miR-NC
2lane:LNCaP miR-29b-3p mimic
3lane:LNCaP miR-29b-3p inhibitor

Fig 3M

M 1 2 3 4 5 6

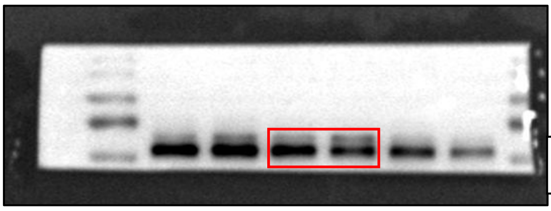

C-Myc (55KD)

3lane:MHCC97H 4mM Gln
4lane:MHCC97H 0mM Gln

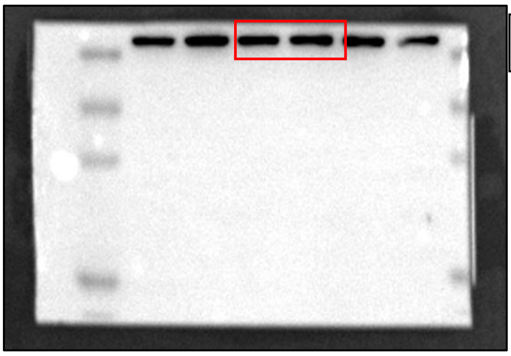

β-actin (43KD)

3lane:MHCC97H 4mM Gln
4lane:MHCC97H 0mM Gln

M 1 2

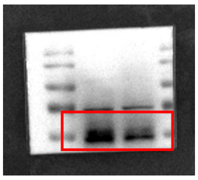

C-Myc (55KD)

1lane:LNCaP 4mM Gln
2lane:LNCaP 0mM Gln

M 1 2

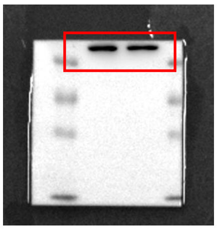

β-actin (43KD)

1lane:LNCaP 4mM Gln
2lane:LNCaP 0mM Gln

Fig 3Q

M 1 2 3 4

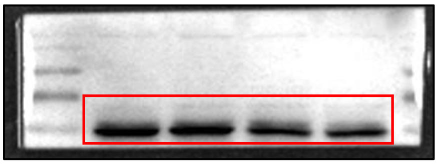

C-Myc (55KD)

1lane:MHCC97H APTO-253 0 mM
2lane:MHCC97H APTO-253 2 mM
3lane:MHCC97H APTO-253 5 mM
4lane:MHCC97H APTO-253 10 mM

M 1 2 3 4

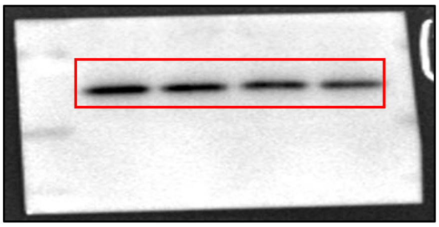

GGCT (21KD)

1lane:MHCC97H APTO-253 0 mM
2lane:MHCC97H APTO-253 2 mM
3lane:MHCC97H APTO-253 5 mM
4lane:MHCC97H APTO-253 10 mM

M 1 2 3 4

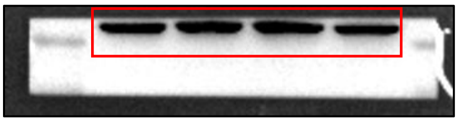

β-actin (43KD)

1lane:MHCC97H APTO-253 0 mM
2lane:MHCC97H APTO-253 2 mM
3lane:MHCC97H APTO-253 5 mM
4lane:MHCC97H APTO-253 10 mM

M 1 2 3 4

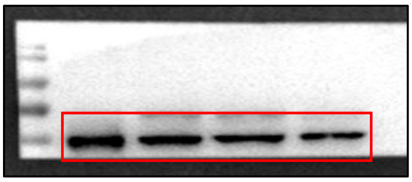

C-Myc (55KD)

1lane:LNCaP APTO-253 0 mM
2lane:LNCaP APTO-253 2 mM
3lane:LNCaP APTO-253 5 mM
4lane:LNCaP APTO-253 10 mM

M 1 2 3 4

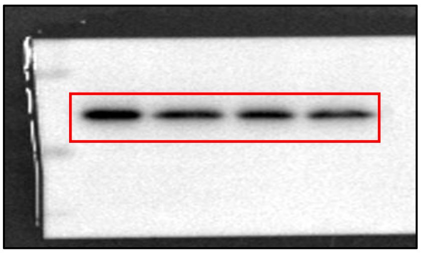

GGCT (21KD)

1lane:LNCaP APTO-253 0 mM
2lane:LNCaP APTO-253 2 mM
3lane:LNCaP APTO-253 5 mM
4lane:LNCaP APTO-253 10 mM

M 1 2 3 4

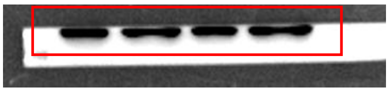

β-actin (43KD)

1lane:LNCaP APTO-253 0 mM
2lane:LNCaP APTO-253 2 mM
3lane:LNCaP APTO-253 5 mM
4lane:LNCaP APTO-253 10 mM

Fig 4E

M 1 2 3

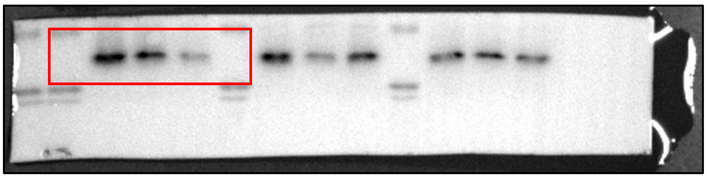

GGCT (21KD)

1lane:MHCC97H Ctrl
2lane:MHCC97H siGGCT162
3lane:MHCC97H siGGCT299

M 1 2 3

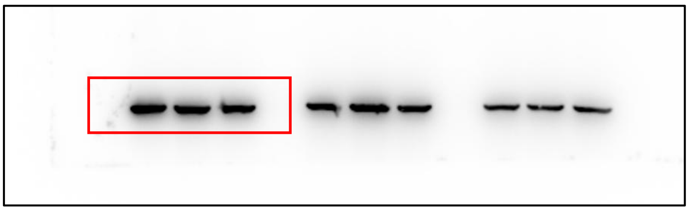

β-actin (43KD)

1lane:MHCC97H Ctrl

2lane:MHCC97H siGGCT162

3lane:MHCC97H siGGCT299

M 1 2 3

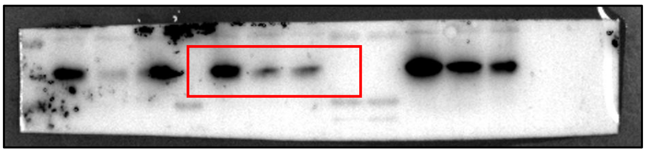

GGCT (21KD)

1lane:HepG2 Ctrl

2lane:HepG2 siGGCT162

3lane:HepG2 siGGCT299

M 1 2 3

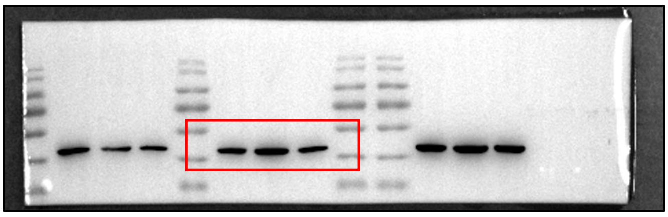

β-actin (43KD)

1lane:HepG2 Ctrl

2lane:HepG2 siGGCT162

3lane:HepG2 siGGCT299

Fig 4G

M 1 2 3 4

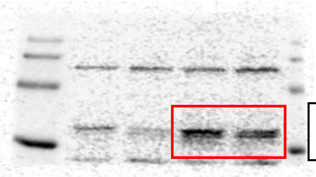

CCNB1 (58KD)

3lane:MHCC97H Ctrl

4lane:MHCC97H siGGCT

M 1 2 3 4

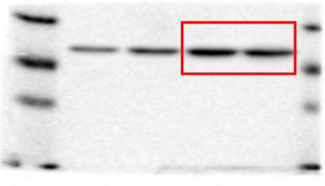

CDK1 (34KD)

3lane:MHCC97H Ctrl
4lane:MHCC97H siGGCT

M 1 2

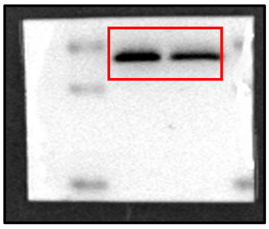

p-CDK1 (34KD)

3lane:MHCC97H Ctrl
4lane:MHCC97H siGGCT

M 1 2 3 4

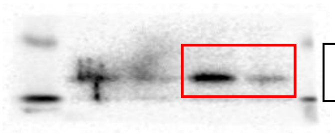

GGCT (21KD)

3lane:MHCC97H Ctrl
4lane:MHCC97H siGGCT

M 1 2 3 4

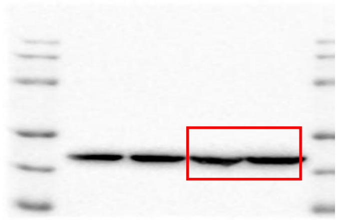

β-actin (43KD)

3lane:MHCC97H Ctrl
4lane:MHCC97H siGGCT

M 1 2 3 4

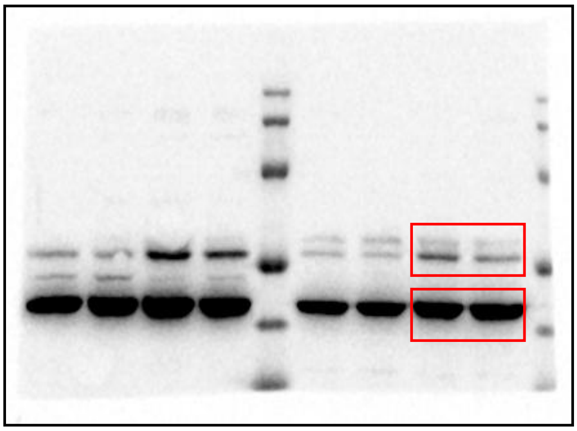

CCNB1 (58KD)

β-actin (43KD)

3lane:HepG2 Ctrl

4lane:HepG2 siGGCT

M 1 2 3 4

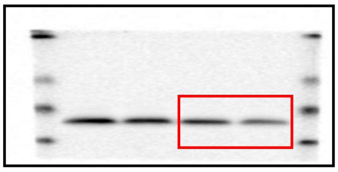

CDK1 (34KD)

3lane:HepG2 Ctrl

4lane:HepG2 siGGCT

M 1 2

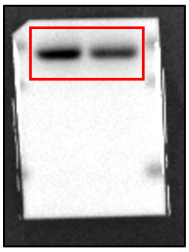

p-CDK1 (34KD)

3lane:HepG2 Ctrl

4lane:HepG2 siGGCT

M 1 2 3 4

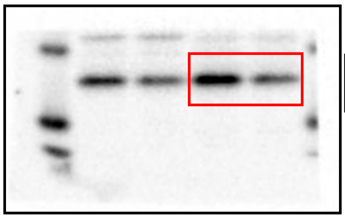

GGCT (21KD)

3lane:HepG2 Ctrl

4lane:HepG2 siGGCT

M 1 2 3 4

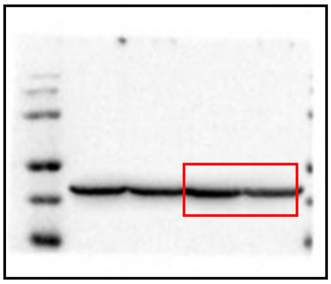

β-actin (43KD)

3lane:HepG2 Ctrl

4lane:HepG2 siGGCT

Fig 5E

M 1 2 3

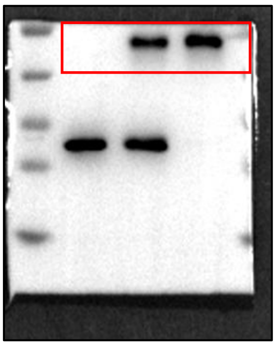

GGCT (ex)(46KD)

1lane:PC3 eGFP

2lane:PC3GGCT-eGFP

3lane:PC3GGCT-E98A-eGFP

M 1 2 3

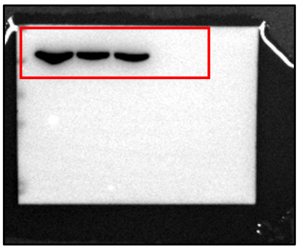

β-actin(43KD)

1lane:PC3eGFP

2lane:PC3GGCT-eGFP

3lane:PC3GGCT-E98A-eGFP

M 1 2 3

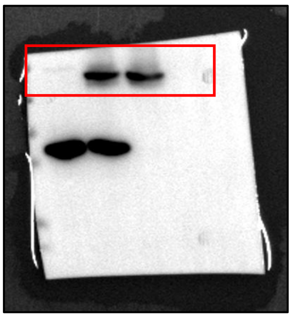

GGCT (ex)(46KD)

1lane:DU145eGFP

2lane:DU145GGCT-eGFP

3lane:DU145GGCT-E98A-eGFP

M 1 2 3

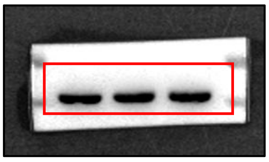

β-actin(43KD)

1lane:DU145eGFP

2lane:DU145GGCT-eGFP

3lane:DU145GGCT-E98A-eGFP

Fig 6I

1 2 3

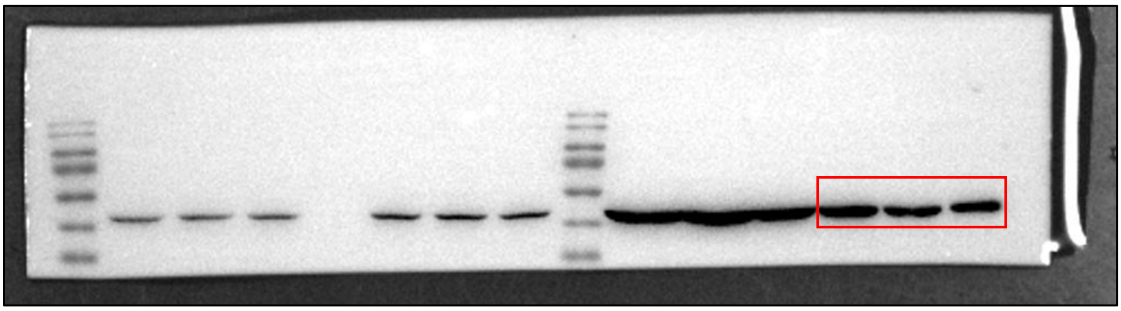

β-actin(43KD)

1lane:ctrl

2lane:siGGCT

3lane:siGGCT/NAC

1 2 3

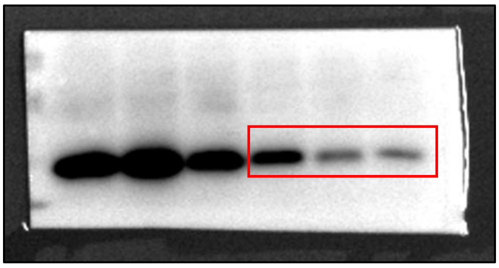

GGCT(21KD)

1lane:ctrl

2lane:siGGCT

3lane:siGGCT/NAC

Fig S1F

1 2

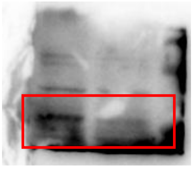

CCNB1 (58KD)

1 lane: LNCaP 4mM Gln

2 lane: LNCaP 0mM Gln

1 2

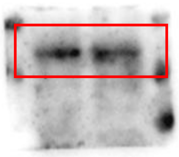

CDK1 (34KD)

1 lane: LNCaP 4mM Gln

2 lane: LNCaP 0mM Gln

1 2

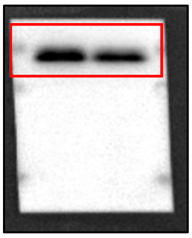

p-CDK1 (34KD)

1 lane: LNCaP 4mM Gln

2 lane: LNCaP 0mM Gln

1 2

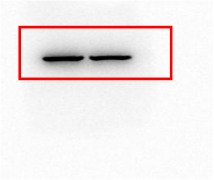

β-actin(43KD)

1 lane: LNCaP 4mM Gln

2 lane: LNCaP 0mM Gln

1 2

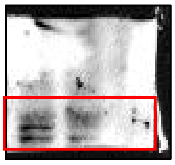

CCNB1 (58KD)

1 lane:C4-2 4mM Gln

2 lane: C4-2 0mM Gln

1 2

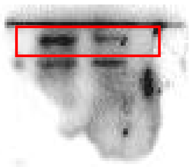

CDK1 (34KD)

1 lane:C4-2 4mM Gln

2 lane: C4-2 0mM Gln

1 2

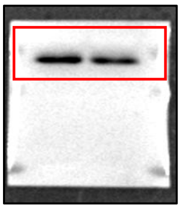

p-CDK1 (34KD)

1 lane:C4-2 4mM Gln

2 lane: C4-2 0mM Gln

1 2

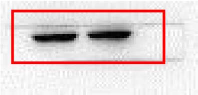

β-actin(43KD)

1 lane:C4-2 4mM Gln

2 lane: C4-2 0mM Gln

Fig S4E

1 2

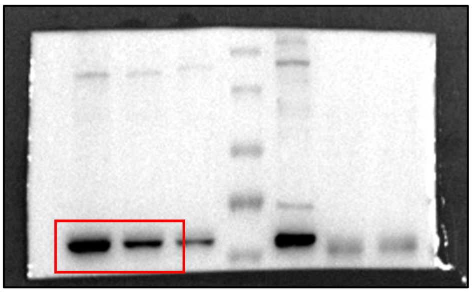

CCNB1 (58KD)

1lane:LNCaP Ctrl

2lane:LNCaP siGGCT

M 1 2

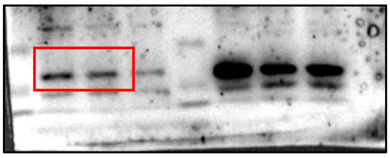

CDK1 (34KD)

1lane:LNCaP Ctrl

2lane:LNCaP siGGCT

M 1 2

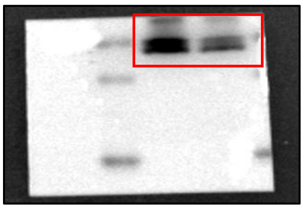

p-CDK1 (34KD)

1lane:LNCaP Ctrl

2lane:LNCaP siGGCT

M 1 2

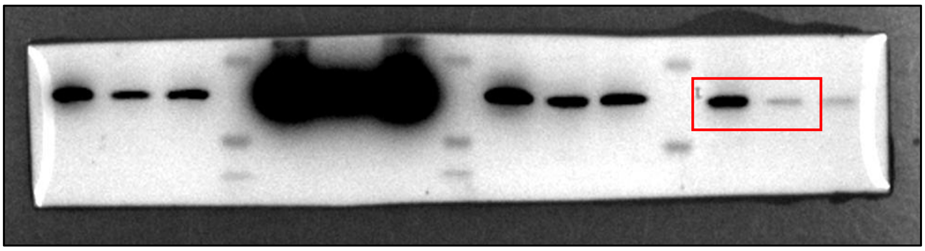

GGCT(21KD)

1lane:LNCaP Ctrl

2lane:LNCaP siGGCT

M 1 2

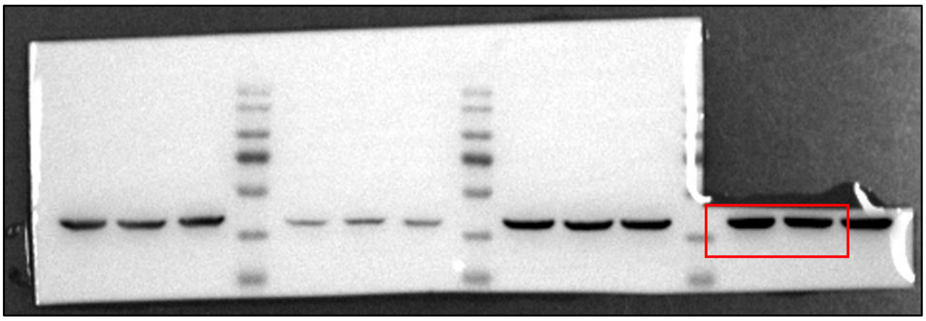

β-actin(43KD)

1lane:LNCaP Ctrl

2lane:LNCaP siGGCT

M 1 2

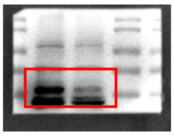

CCNB1 (58KD)

1lane:C4-2 Ctrl

2lane:C4-2 siGGCT

M 1 2

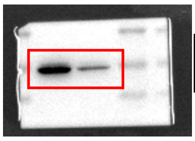

CDK1 (34KD)

1lane:C4-2 Ctrl

2lane:C4-2 siGGCT

M 1 2

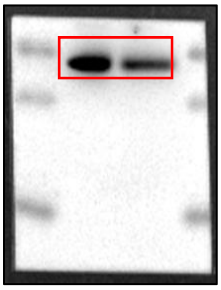

p-CDK1 (34KD)

1lane:C4-2 Ctrl

2lane:C4-2 siGGCT

M 1 2

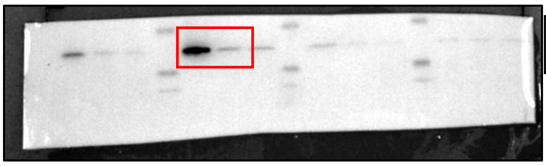

GGCT(21KD)

1lane:C4-2 Ctrl

2lane:C4-2 siGGCT

M 1 2

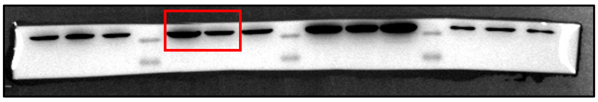

β-actin(43KD)

1lane:C4-2 Ctrl

2lane:C4-2 siGGCT
